# Starvation-induced changes in somatic insulin/IGF-1R signaling drive metabolic programming across generations

**DOI:** 10.1126/sciadv.ade1817

**Published:** 2023-04-07

**Authors:** Merly C. Vogt, Oliver Hobert

**Affiliations:** Department of Biological Sciences, Howard Hughes Medical Institute, Columbia University, New York, NY 10027, USA.

## Abstract

Exposure to adverse nutritional and metabolic environments during critical periods of development can exert long-lasting effects on health outcomes of an individual and its descendants. Although such metabolic programming has been observed in multiple species and in response to distinct nutritional stressors, conclusive insights into signaling pathways and mechanisms responsible for initiating, mediating, and manifesting changes to metabolism and behavior across generations remain scarce. By using a starvation paradigm in *Caenorhabditis elegans*, we show that starvation-induced changes in dauer formation-16/forkhead box transcription factor class O (DAF-16/FoxO) activity, the main downstream target of insulin/insulin-like growth factor 1 (IGF-1) receptor signaling, are responsible for metabolic programming phenotypes. Tissue-specific depletion of DAF-16/FoxO during distinct developmental time points demonstrates that DAF-16/FoxO acts in somatic tissues, but not directly in the germline, to both initiate and manifest metabolic programming. In conclusion, our study deciphers multifaceted and critical roles of highly conserved insulin/IGF-1 receptor signaling in determining health outcomes and behavior across generations.

## Teaser

Diet-induced changes in somatic insulin signaling mediate adverse physiological and behavioral outcomes in subsequent generations.

## INTRODUCTION

One of the major health challenges many societies are facing today is the rampant global increase in obesity and associated comorbidities across all age groups. In addition to distinct genetic predispositions and a shift to poor nutrition and sedentary lifestyles, increasing evidence shows that the nutritional experiences of our ancestors have a strong impact on our susceptibility to develop these diseases. The concept that an adverse nutritional and metabolic environment can exert intergenerational and potentially transgenerational effects on metabolism and health is often referred to as “metabolic programming” (*1*–*4*).

Some of the most well-known and highly referenced human epidemiological datasets that have been instrumental in our current understanding of metabolic programming described the long-term health outcomes of children whose mothers were exposed to the so-called “Dutch Hunger Winter” between 1944 and 1945. An embargo on food supplies to the northern regions of the Netherlands forced severe food rationing and resulted in a substantial reduction of caloric intake. This severe nutrient restriction was particularly impactful for pregnant women. Because of the clearly defined time period of nutrient deprivation, retrospective studies indicated that exposure to such an abnormal nutritional environment during pregnancy resulted in higher incidences of metabolic impairments, including obesity, glucose intolerance, hyperlipidemia, and cardiovascular diseases in the next generation (*5*–*10*). An elevated body weight and body mass index was also reported for grandchildren of women that experienced the Dutch Hunger Winter during pregnancy (*11*, *12*). Apart from the effects on the descendants’ metabolic health, subsequent studies further suggested a link between gestational nutrient deprivation and an increased risk to develop psychiatric disorders, such as schizophrenia, antisocial personality disorder, and major affective disorder (*13*, *14*). Similar associations between famine exposure and the development of mental illnesses were observed in studies examining the long-term health outcome of children exposed to the Chinese famine (1959–1961) in utero or during early postnatal life (*15*–*18*). Together, these studies highlight the complex and potentially detrimental long-term impact of malnutrition on metabolic and mental health of subsequent generations.

In light of the ever-growing incidences of obesity and diabetes, particularly in women of child-bearing age (*19*), current research mainly focuses on understanding the impact of parental overnutrition on the health outcomes of the subsequent generations. Similar metabolic, cardiovascular, and even neurological impairments described for children of undernourished mothers were reported as a result of maternal obesity, diabetes, and overnutrition (*20*–*26*). These observations have been confirmed by animal models of metabolic programming using not only a wide range of nutritional or metabolic stressors but also different species, including nonhuman primates, sheep, mice, flies, nematodes, and daphnia (*27*–*43*). The strikingly similar effects of parental diet and nutrition on descendants’ health outcomes across species and stressors suggest potentially highly conserved signals and molecular mechanisms underlying metabolic programming.

Animal models of metabolic programming have provided insights into the extent and magnitude of effects of parental diet on the cellular and molecular profile, as well as function of tissues critical in the regulation of metabolism (e.g., white adipose tissue, skeletal muscle, and nervous system) across generations (*44*–*47*). However, causative links between diet-induced metabolic, cellular, molecular, and even epigenetic changes observed in the parental generations and the development and manifestation of metabolic and behavioral changes in subsequent generations remain scarce. To advance our understanding of mechanisms underlying metabolic programming, it is therefore critical to identify the key signals that are causative to and not simply a side effect of adverse metabolic and behavioral phenotypes in each generation. The current lack of mechanistic insight is likely due to the complex nature of intergenerational and especially transgenerational metabolic programming paradigms, in which the distinct role(s) of such changes has to be dissected not only on a systemic but also on a generational level. Whereas intergenerational effects could occur as direct consequences of stress exposure via the germline (i.e., F1), effects are considered to be transgenerational, when phenotypes are present in generations that have never directly been exposed to the original trigger (>F1 or even >F2, depending on paradigm).

Thus, using a relatively simple organism that allows for an in-depth and systematic dissection of the relative contribution and the spatiotemporal dynamics of cellular and molecular processes underlying metabolic programming could lead to fundamental insights into how differences in disease susceptibility are established early in life. The nematode *Caenorhabditis elegans* provides distinct advantages that allows for such an approach. First, its short generation time and high brood size considerably facilitate identifying and studying inter- and transgenerational phenotypes. Second, *C. elegans* can survive long periods of nutrient deprivation, which has previously been reported to result in some molecular and physiological changes across generations (*40*, *42*, *43*). Third, *C. elegans* exhibits complex behaviors that depend on highly conserved pathways regulating metabolism, such as the insulin/insulin-like growth factor 1 (IGF-1) receptor signaling (IIS), adenosine monophosphate–activated protein kinase (AMPK), transforming growth factor–β (TGF-β), and serotonergic [5-hydroxytryptamine (5-HT)] pathways. Last, because of its amenability to genetic manipulation, signaling pathways can be disrupted with temporal and spatial specificity (*48*–*50*), thereby allowing to dissect molecular mechanisms of animal physiology and behavior.

Because of these advantages, we established a starvation paradigm in *C. elegans* and found that early life starvation results in robust transcriptomic, physiological, and behavioral changes intragenerationally, intergenerationally, and even transgenerationally. Moreover, we show that early life starvation–induced activation of dauer formation-16/forkhead box transcription factor class O (DAF-16/FoxO) activity, the main downstream target of IIS (*51*), persists into adulthood even in the absence of acute environmental stress. Tissue-specific depletion of DAF-16/FoxO during distinct developmental time points demonstrates that DAF-16/FoxO acts in somatic tissues, but not directly in the germline, to initiate metabolic programming in the parental generation, and further manifests the development of adverse behavioral and metabolic phenotypes in subsequent generations. Last, we show that intertissue IIS from the intestine and nervous system contributes to defects in fecundity in the parental generation, as well as reduced stress resistance in their descendants. In conclusion, we provide direct evidence that nutrition-induced changes in IIS in somatic tissues are critical for driving metabolic programming inter- and transgenerationally. Moreover, the striking similarities in phenotypes observed as a result of parental malnutrition in this study and mammalian models of metabolic programming support the notion that metabolic programming is highly conserved and further validate the use of *C. elegans* as a first step to finally advance our understanding of the underlying mechanisms responsible for mediating changes in disease susceptibility across generations.

## RESULTS

### Early life starvation of ancestors increases the exploratory behavior of well-fed descendants inter- and transgenerationally

**Fig. 1.**
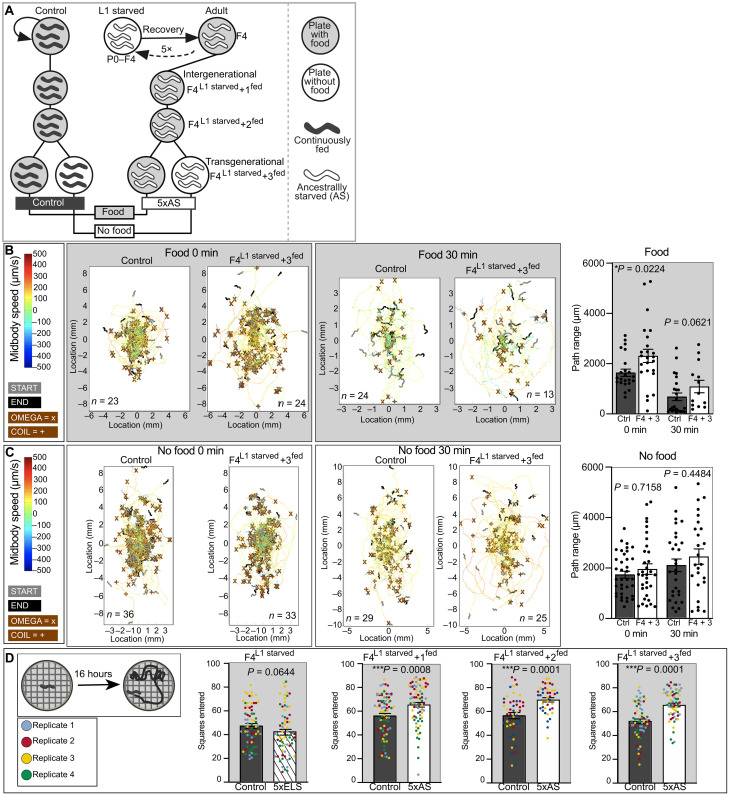
Early life starvation increases exploratory behavior inter- and transgenerationally in a food-dependent manner. (**A**) Schematic overview of experimental outline. Gray circle, food; white circle, no food; dark gray worms, continuously fed controls; white worms, animals that experienced L1 starvation in parental generation. (**B**) Automated single-worm tracking analysis of control and F4^L1starved^ + 3^fed^ revealed increased exploratory behavior in ancestrally starved animals under fed conditions at the L4 stage. (**C**) Exploratory behavior was not different between control and F4^L1starved^ + 3^fed^ when tracked in the absence of food. The same animals were tracked after being placed on tracking plate after 0 and 30 min. The explored path of each animal is visualized from its starting position (gray worm) to its end point (black worm) within a 5-min time frame. All animals within the same group under the same condition are plotted in the same graph, which also includes the average midbody speed (μm/s) and position of all omega turns (X) and coils (+) for each animal. The total path range (μm) of each animal was used for statistical analysis (Mann-Whitney *U* test). Data were obtained from three independent biological replicates (starting with *n* = 10 per replicate on food and *n* = 15 per replicate on no food) and is presented as mean ± SEM. Each dot represents one animal. (**D**) An independent experimental setup confirmed increased exploratory behavior in descendants of early life starved animals over a 16-hour period (start of experiment with young L4 animals). Data were obtained from three to four independent biological replicates (indicated by different colors of each data point) with *n* = 15 to 20 animals per replicate. Data are presented as mean ± SEM. Significance was determined by Mann-Whitney *U* test. See also fig. S1. 5xELS, 5× early-life starved. 5xAS, 5× ancestrally starved.

To shed light on the molecular mechanisms and long-term consequences of metabolic programming, we first established a recurrent starvation paradigm in *C. elegans* (Fig. 1A). When hatching in the absence of food, *C. elegans* larvae arrest in the early juvenile (L1) stage and undergo substantial molecular adaptations to survive during this period (*52*). However, L1-arrested animals do not undergo gross phenotypical changes and are able to resume development upon encountering food sources, thus presenting a powerful model to study the inter- and transgenerational effects of early life malnutrition (*40*–*42*). We sought to maximize exposure to the original nutritional stressor by exposing wild-type L1-stage animals to starvation for 6 days during five consecutive generations (P0^L1starved^ to F4^L1starved^). Subsequently, we kept the next two generations under well-fed conditions (F4^L1starved^ + 1^fed^ and F4^L1starved^ + 2^fed^), and we then analyzed the following generation (F4^L1starved^ + 3^fed^) under well-fed or acute starvation conditions for robust behavioral, physiological, and molecular phenotypes. Control animals remained well fed throughout the paradigm unless acute starvation was required for specific experiments (Fig. 1A).

We reasoned that recurrent L1 starvation would maximize the animals’ exposure to the nutritional stressor and potentially result in more robust and reliable phenotypes in subsequent generations, after the initial stressor has vanished. Reexposure to the same stressor, including diet and heat, over multiple generations had previously been shown to lead to more robust phenotypes inter- and transgenerationally (*53*–*55*). Moreover, we focused our initial analysis on the great-grandchildren of L1-starved animals (F4^L1starved^ + 3^fed^) to identify phenotypes that are truly transgenerational in nature. In contrast to intergenerational effects that could occur as direct consequences of stress exposure via the germline, transgenerational effects are seen in a generation that has never directly been exposed to the original trigger. We anticipated that such setup would provide robust readouts to identify the signals and mechanisms underlying metabolic programming across generations.

As a first unbiased screen for long-term metabolic programming in *C. elegans*, we used an automated single-worm tracker that measures animal locomotory and exploratory behavior (*56*) to analyze control and F4^L1starved^ + 3^fed^ animals in the presence of food, as well as in response to acute no food conditions. Overall, we did not detect any gross or consistent changes in morphology or posture as a consequence of ancestral early life starvation. However, within the first 5 min of being placed on a tracking plate with food, F4^L1starved^ + 3^fed^ animals explored a larger area of this environment compared to control animals during that time frame (Fig. 1B). This result suggests that repeated starvation in ancestral generations could determine the pattern of exploratory behavior of well-fed descendants’ multiple generations later.

Depending on food availability and quality, *C. elegans* adapts the time spent between two behavioral states, i.e. roaming and dwelling, over time (*57*, *58*). To identify any potential changes in this adaptive behavior, we analyzed the same animals a second time 30 min after being placed on the tracking plate. We found that the path range for both control and F4^L1starved^ + 3^fed^ animals was reduced relative to the first time point, as expected. Although the difference in path range between these groups at this later time point did not reach statistical significance (*P* = 0.0621), more of the ancestrally starved (*n* = 8 of 30) than control animals (*n* = 2 of 30) had moved out of frame during the experiment and thus were automatically excluded from the tracking analysis. This experimental limitation might have masked real and prolonged increases in exploratory behavior in F4^L1starved^ + 3^fed^ animals in the presence of food.

In contrast to their behavior on food, we did not detect differences in path range or any other changes in morphological or behavioral features between control and F4^L1st^ + 3^fed^ animals when tracked in the absence of food (Fig. 1C). Together, the results of the tracking analysis demonstrate that early life malnutrition modestly affects exploratory behavior in a food-dependent manner transgenerationally.

To validate this finding, we assessed the exploratory behavior of early life starved animals across generations in a different experimental setup, which determines the area explored over a 16-hour period. Early life starvation significantly increased exploratory behavior of their progeny (F4^L1starved^ + 1^fed^), as well as in subsequent generations (F4^L1starved^ + 2^fed^, F4^L1starved^ + 3^fed^), independently confirming our findings described above (Fig. 1D). Moreover, these results suggest that ancestrally starved animals display not only an increased initial exploration of a novel environment but also a long-term behavioral change. We found that animals that experienced starvation during early life (F4^L1starved^) did not show the same increase but rather a trend toward reduced exploration compared to control animals (*P* = 0.0644; Fig. 1D). This opposing effect of early life versus ancestral starvation on the animals’ exploratory behavior could point to distinct signals and mechanisms affecting the parental generation compared to their descendants.

We observed the same pattern of exploratory changes across generations after only one round of L1 starvation (P0^L1starved^, P0^L1starved^ + 1^fed^, P0^L1starved^ + 2^fed^, and P0^L1starved^ + 3^fed^; fig. S1, A and B). However, one-time ancestral L1 starvation was not sufficient to consistently observe this phenotype across different experimental setups, which validates our original rationale of using a recurrent starvation paradigm. For the remainder of this study, we therefore decided to focus on the more robust recurrent exposure paradigm (Fig. 1A).

### Early life starvation impacts on IIS across generations

**Fig. 2.**
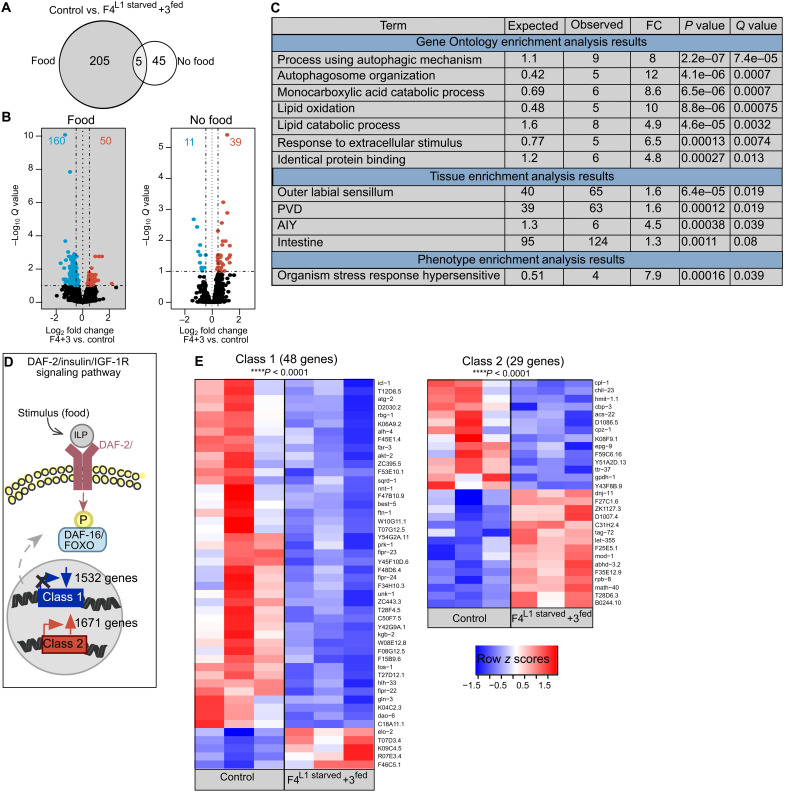
Early life starvation affects IIS signaling across generations. (**A**) Transcriptome analysis of control and F4^L1starved^ + 3^fed^ animals under food and no food condition revealed significant differences in expression of 255 genes total (adjusted *P* < 0.1; Benjamini and Hochberg correction on *P* values obtained by Wald test; *n* = 3 with ~10,000 animals per replicate) in L1 animals. (**B**) Volcano plot of differentially expressed genes between control and F4^L1starved^ + 3^fed^ animals under fed and starved conditions. Blue, relative down-regulation; red, relative up-regulation in F4^L1starved^ + 3^fed^ animals. (**C**) Enrichment analyses of genes differentially expressed between control and F4^L1starved^ + 3^fed^ animals under fed conditions. FC, fold change. (**D**) Overview of IIS pathway in *C. elegans*. ILP, insulin-like peptides. (**E**) Heatmaps depicting relative expression of class 1 and class 2 downstream targets of IIS differentially expressed between control and F4^L1starved^ + 3^fed^ animals. Relative expression of each gene across biological replicates in controls and F4^L1starved^ + 3^fed^ is displayed as row *z* scores in heatmaps. Rows, genes; columns, replicates. Significant enrichment was determined by hypergeometric distribution. See also fig. S2 and data S1 and S5.

To decipher the molecular basis of a nutrition-dependent increase in exploratory behavior in the progeny and subsequent generations of early life starved animals, we compared how control or F4^L1starved^ + 3^fed^ animals alter their transcriptome either while being well fed or after being exposed to acute starvation (data S1). A total of 255 transcripts (adjusted *P* < 0.1) were differentially expressed between these groups across three independent biological replicates (~10,000 animals per replicate). A total of 210 and 50 genes displayed significantly different expression under food and no food conditions, respectively (Fig. 2, A and B, and tables S2 and S3). All five genes (i.e., *abhd-3.2*, *B0272.4*, *C31H2.4*, *dhs-6*, and *dif-1*) significantly different between control and F4^L1st^ + 3^fed^ animals under both conditions are involved in the regulation of lipid metabolism, pointing to transgenerational changes in metabolism in response to early life starvation (Fig. 2C and fig. S2A).

We found that a large number of differentially expressed genes were downstream targets of the highly conserved IIS pathway, one of the main signaling pathways that regulate metabolism across species (data S4 and S5) (*59*). In *C. elegans*, stimuli, such as food, lead to the release and subsequent binding of agonistic insulin-like peptides to the DAF-2/insulin/IGF-1 receptor (IIR), which in turn leads to a cascade of phosphorylation events ultimately resulting in the inhibitory phosphorylation and subsequent exclusion of the DAF-16/FoxO transcription factor from the nucleus (Fig. 2D) (*51*, *60*–*64*). This signaling cascade leads to an overall down-regulation of 1532 DAF-16/FoxO–dependent (“class 1”) and overall up-regulation of 1671 DAF-16/FoxO–independent (“class 2”) genes (Fig. 2D) (*65*). Within the set of differentially expressed genes under food conditions, we found a significant enrichment for both class 1 (48 of 210; *P* < 0.0001) and class 2 (29 of 210; *P* < 0.0001) genes, with the majority of class 1 genes relatively down-regulated in F4^L1starved^ + 3^fed^ animals compared to controls (Fig. 2E). We did not find any significant enrichment for genes regulated by other signaling pathways that control metabolism, such as TGF-β (7 of 210) (*66*), serotonin (4 of 210) (*67*), or AMPK (2 of 210) (*68*) signaling pathways. In conclusion, these findings could suggest that early life starvation leads to an increased basal activity of IIS in subsequent generations in the presence of food, resulting in the observed behavioral and transcriptional phenotypes. This hypothesis is in line with a previous study demonstrating that IIS regulates the amount of time animals spend roaming versus dwelling and that loss of DAF-2/IIR function results in decreased roaming compared to control animals in the presence of food (*58*).

**Fig. 3.**
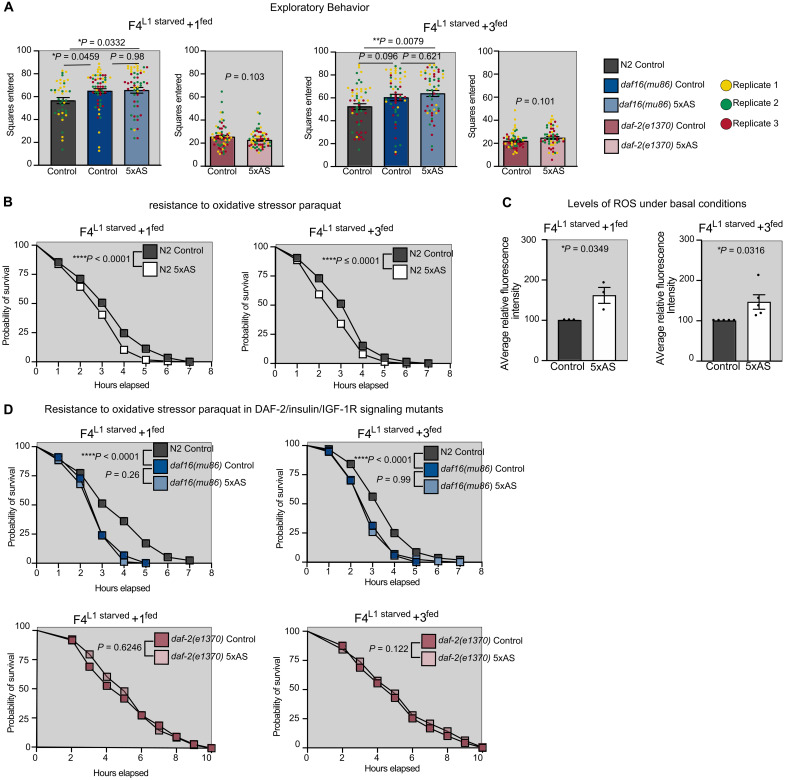
Aberrant IIS is responsible for inter- and transgenerational changes in behavior and stress resistance following early life starvation. (**A**) Inter- and transgenerational changes in exploratory behavior depend on IIS. Data were obtained from three independent biological replicates (indicated by different colors of each data point) with *n* = 15 to 20 animals per replicate. Exploratory behavior was determined after 16 hours (start of experiment with young L4 animals). Data are presented as mean ± SEM. Significance was determined by Mann-Whitney *U* test. (**B**) L1 starvation impaired resistance to oxidative stressor paraquat inter- and transgenerationally in adult animals. (**C**) Ancestrally starved animals displayed increased levels of ROS under basal conditions at the L1 stage. Data are displayed as mean ± SEM from average fluorescence over four time points relative to control animals from three to five independent biological replicates with *n* = 10 to 20 per replicate. Statistical significance was determined by unpaired Student’s *t* test. (**D**) Decreased oxidative stress resistance in ancestrally starved animals depends on IIS. Data were obtained in animals at day 2 of adulthood. Oxidative stress resistance is presented as combined data of two to four independent replicates with 80 to 100 animals per replicate. Significance was determined by log rank (Mantel-Cox test).

To test whether the observed increased exploratory phenotype of ancestrally starved animals was due to an increased basal activity of IIS transgenerationally, we exposed DAF-2/IIR [*daf-2(e1370)*] (*61*) and DAF-16/FoxO [*daf-16(mu86)*] (*51*) loss-of-function mutants to our recurrent starvation paradigm and assayed their exploratory behavior. Loss of DAF-16/FoxO function resulted in a slight increase, whereas loss of DAF-2/IIR function resulted in a significant reduction of exploration in control animals compared to wild-type N2 control animals (Fig. 3A). Loss of DAF-2/IIR or DAF-16/FoxO function may disrupt the small increase in exploratory behavior we observed in Fig. 1 between wild-type control and F4^L1starved^ + 1^fed^, as well as F4^L1starved^ + 3^fed^ animals (Fig. 3A). These results suggest that inter- and transgenerational increased exploratory behavior is mediated by changes in IIS activity following ancestral starvation.

On the basis of these results and our enrichment analysis, we further tested whether ancestral starvation might affect other IIS-dependent phenotypes in *C. elegans*. Despite the highly conserved role of IIS in regulating life span and previous studies suggesting that early life starvation (L1 or dauer) increased life span transgenerationally (*42*, *43*), we were unable to detect such effects on life span after either 1× or 5× ancestral starvation under normal growth conditions (fig. S2B). However, when challenged with high levels of the oxidative stress–inducing chemical paraquat (*69*, *70*) and in support of our hypothesis, F4^L1starved^ + 1^fed^ and F4^L1starved^ + 3^fed^ N2 wild-type animals displayed significantly reduced resistance to oxidative stress compared to control wild-type animals (Fig. 3B). This hypersensitivity to oxidative stress of ancestrally starved animals was accompanied by elevated levels of reactive oxygen species (ROS) under basal conditions (Fig. 3C). Moreover, we found that reduced stress resistance was due to changes in the basal activity of IIS: As expected, loss of DAF-16/FoxO decreased and loss of DAF-2/IIR function increased oxidative stress resistance in control mutant animals compared to control wild-type animals, respectively. Both loss-of-function mutants reverted the differences between those control, F4^L1starved^ + 1^fed^, as well as F4^L1starved^ + 3^fed^ mutant animals (Fig. 3D), suggesting that the previously observed differences between those groups occur likely as a result of aberrant IIS in 5× ancestrally-starved (5xAS) animals. Together, results from our transcriptomic, behavioral, and physiological assays demonstrate that early life starvation results in increased basal activity of DAF-2/IIR inter- and transgenerationally. This change in metabolic activity impairs the ability of the animals to respond to external stimuli, such as stress, and results in prolonged exploration of the environment even though food is present.

### L1 starvation has long-lasting effects on signaling pathways regulating metabolism

**Fig. 4.**
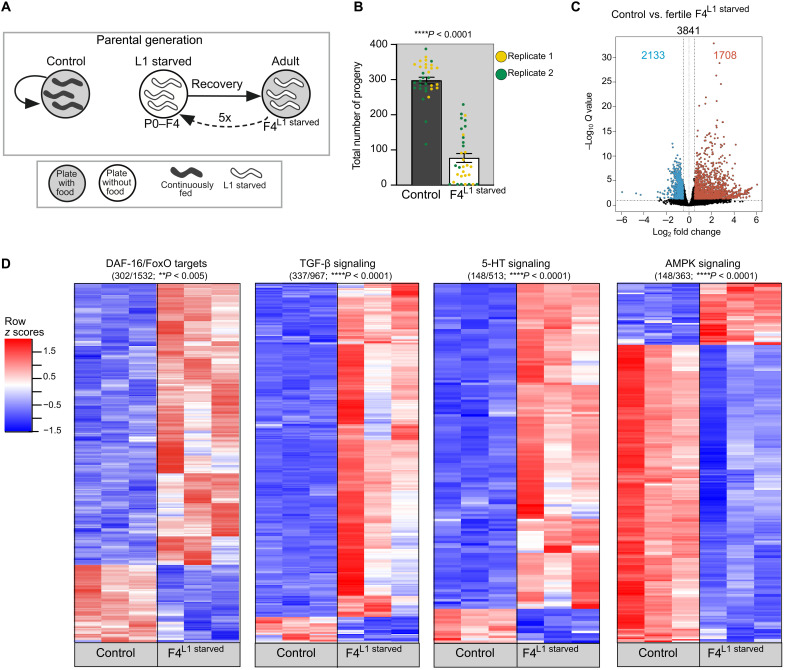
L1 starvation has long-lasting effects on signaling pathways regulating metabolism. (**A**) Schematic overview of parental generation. Gray circle, food; white circle, no food; dark gray worms, continuously fed controls; white worms, animals that experienced L1 starvation. (**B**) L1 starvation decreased brood size of adult animals. Data were obtained from two independent biological replicates (indicated by different colors of each data point) with *n* = 16 animals per replicate. Data are presented as mean ± SEM. Significance was determined by Mann-Whitney *U* test. (**C**) Volcano plot of differentially expressed genes between control and fertile F4^L1starved^ animals under fed conditions (adjusted *P* < 0.05; Benjamini and Hochberg correction on *P* values obtained by Wald test; *n* = 3 with ~100 animals per replicate). Blue, relative down-regulation; red, relative up-regulation in fertile F4^L1starved^ animals. (**D**) L1 starvation affected expression of genes downstream of signaling pathways regulating metabolism, i.e., DAF-16/FoxO, TGF-β, serotonergic (5-HT), and AMPK signaling, in adulthood. Relative expression of each gene across biological replicates in controls and fertile F4^L1starved^ is displayed as row *z* scores in heatmaps. Values were *z* score normalized and plotted using heatmap3 in RStudio. Each row represents a single gene (adjusted *P* ≤ 0.05), and each column represents a single RNA-sequencing replicate. Blue, relative down-regulation; red, relative up-regulation in fertile F4^L1starved^ animals. Significant enrichment was determined by hypergeometric distribution. See also fig. S3 and data S7 to S12.

To understand how such an increase in IIS activity is mediated across generations, we focused our analysis on the parental generation that experienced the nutritional stressor during early life (Fig. 4A). Like control wild-type animals, the majority of L1-starved animals reached adulthood 3 days after reexposure to food and did not show any gross developmental delays. However, prolonged exposure to starvation during the L1 stage significantly decreased the number of progeny compared to control animals, albeit not to the same extent across the population (Fig. 4B). About 25% of L1-starved animals did not produce any progeny, whereas the most fertile 25% of the population was able to produce a brood between 158 and 227 animals (compared to ~300 of nonstarved animals). This range of fertility defects highlighted an intrinsic variability of an isogenic population in their ability to respond to and recover from nutritional stress exposure during development. Because of the nature of our starvation paradigm, the majority of animals that form the next generations inevitably originate from the most fertile fraction of the population.

Thus, to obtain insights into how early life starvation affects IIS activity in subsequent generations, we analyzed the transcriptome of currently fed, manually picked, clearly distinguishable fertile day-1-adult F4^L1starved^ (>5 eggs) and sterile day-1-adult F4^L1starved^ animals (without eggs), as well as continuously fed control animals (data S6). A total of 3841 transcripts were significantly different (adjusted *P* < 0.05) across three independent biological replicates (*n* = 100 animals per replicate) between control and F4^L1starved^ animals that were able to produce progeny and thus contributed to the next generation (“fertile F4^L1starved^”) (Fig. 4C and data S7). Among these genes, we found a significant enrichment and overall up-regulation of class 1 genes downstream of the DAF-16/FoxO transcription factor (*65*). Moreover, a significant proportion of genes down-regulated in TGF-β– and 5-HT–signaling loss-of-function mutants were up-regulated (*66*, *67*), while a significant proportion of genes down-regulated in AMPK signaling loss-of-function mutants were down-regulated in fertile, fed adult animals that experienced L1 starvation compared to control animals (Fig. 4D and data S8 to S11) (*68*). These results demonstrate that starvation during a very specific developmental time window is able to exert molecular and likely metabolic changes that last into the reproductive adult stage even under well-fed conditions.

### Starvation-induced nuclear translocation of DAF-16/FoxO in the germline persists into adulthood

**Fig. 5.**
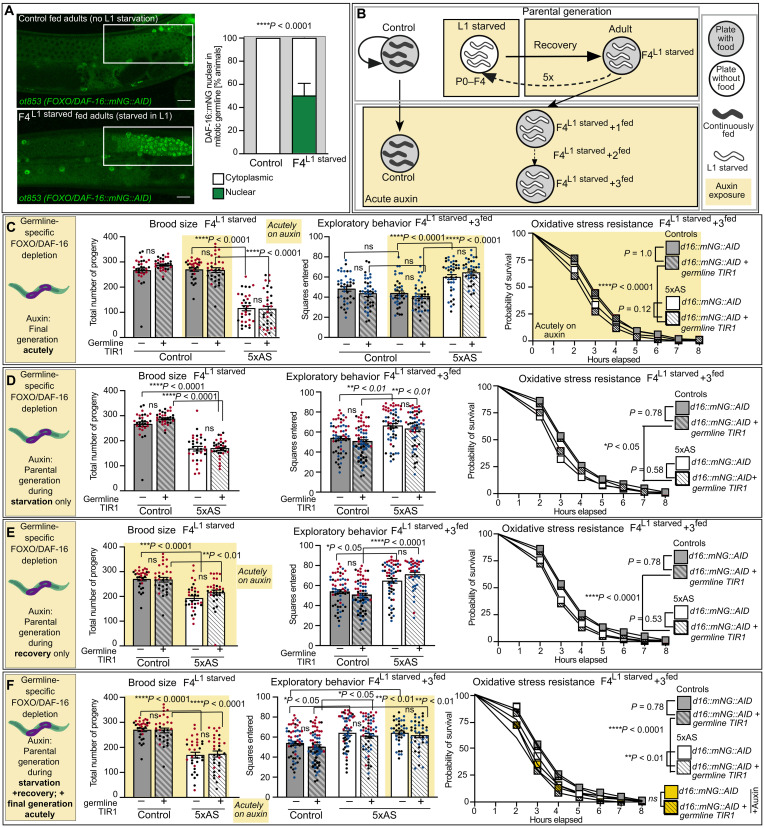
DAF-16/FoxO does not act directly in the germline to mediate metabolic programming across generations. (**A**) CRISPR-Cas-9–generated endogenous DAF-16/FoxO::mNeonGreen translational reporter revealed prominent nuclear accumulation of DAF-16/FOXO in mitotic germline of L1-starved, adult animals under fed conditions. Data were obtained from four independent biological replicates with *n* = 10 per replicate at day 1 of adulthood. Data are presented as mean ± SEM. Significance was determined by chi-square test. Color indicates subcellular localization of DAF-16/FoxO in mitotic germline: White, cytoplasm; green, nucleus. Scale bar, 15 μm. (**B**) Experimental outline used for spatiotemporal depletion of DAF-16/FoxO using the AID/TIR1 system. Yellow, temporally restricted exposure to auxin; gray circle, on food; white circle, no food; gray worms, consistently fed worms; white worms, L1 starved in P0-F4. (**C**) Acute germline-specific depletion of DAF-16 did not affect brood size in controls or exploratory behavior and oxidative stress resistance inter- and transgenerationally. Auxin-dependent depletion of DAF-16/FoxO::mNG::AID in the germline via germline-specific TIR1 expression during (**D**) L1 starvation only, (**E**) recovery only, or (**F**) starvation and recovery did not revert defects in brood size in parental generation, or increased exploratory behavior and decreased oxidative stress resistance of subsequent generations. All groups were exposed to auxin during the specific time window as indicated in the left of each panel. A yellow background indicates additional acute exposure to auxin during experiment. For all brood size experiments, data were obtained from two independent biological replicates with *n* = 16 per replicate, each dot representing one animal. Data are presented as mean ± SEM, and significance was determined by one-way ANOVA with post hoc Tukey. For all exploratory assays, data were obtained from two to three independent biological replicates with *n* = 20 per replicate and is displayed as mean ± SEM. Significance was determined by one-way ANOVA with post hoc Tukey. Data for all oxidative stress experiments are presented as combined data from two to three independent biological replicates with *n* = 70 to 96 per replicate. Significance was determined by log rank (Mantel-Cox test). See also fig. S4. ns, not significant.

Acute L1 starvation results in nuclear translocation of DAF-16/FoxO in tissues across the entire animal, including the intestine, nervous system, epidermis, and germline precursor cells, resulting in significant up-regulation of downstream target genes (fig. S4A) (*71*). As a first indication for which tissues are responsible for the continued up-regulation of DAF-16/FoxO target genes into adulthood, we imaged control and F4^L1starved^-fed adult animals expressing an endogenously mNeonGreen-tagged (mNG) *daf-16/foxo* allele (*daf-16/FoxO::mNG; ot853*) (*48*). In line with previous reports, we found it difficult to properly and reliably quantify tissue-specific relatively mild changes in nuclear and somatic DAF-16/FoxO levels between control and F4^L1starved^, currently fed, animals. However, we observed a prominent and robust nuclear accumulation of DAF-16/FoxO specifically in the mitotic germline of currently fed, adult fertile F4^L1starved^ compared to control adults (Fig. 5A). Although acute starvation in the adult stage also led to a more perinuclear enrichment of the DAF-16/FoxO::mNG::AID protein compared to well-fed adults (fig. S4B), the effect of early life L1 versus acute starvation on DAF-16/FoxO::mNG expression and subcellular localization in the mitotic germline was clearly distinguishable. Because of their potential impact on future generations, changes in the germline are of particular interest when examining the effects of parental experiences on health outcomes of subsequent generations. Thus, to conclusively determine how changes in IIS in the germline contribute to metabolic programming across generations, we used the “AID/TIR1 system” to disrupt DAF-16/FoxO function in a spatial and temporally controlled manner (*72*).

To this end, we used a conditional *daf-16/FoxO::mNG* allele (*ot853*) that contains an auxin-inducible degron (AID) at the C terminus. Simultaneous tissue-specific expression of the substrate-recognizing subunit of the plant-specific ubiquitin ligase TIR1 and addition of the substrate auxin allows for robust depletion of DAF-16/FoxO as previously described (fig. S5, A to D) (*48*). Each assay was performed with two strains, of which one only expressed the AID-tagged *daf-16/FoxO::mNG* allele (*ot853*), while the other strain expressed both, *daf-16/FoxO::mNG* and a germline-specific TIR1 transgene (*ot853; ieSi38*). Both strains were exposed to our recurrent starvation paradigm, and DAF-16/FoxO was depleted by temporally controlled addition of water-soluble auxin during four distinct periods: (i) acutely during the final stage of any assay performed; (ii) during L1 starvation only; (iii) during recovery after starvation until adulthood; or (iv) continuously during both, L1 starvation and recovery in F4^L1starved^ animals (Fig. 5B). The strain that did not express TIR1 served as a control for all conditions to exclude any side effects of exposure to auxin itself.

Exposure to auxin alone or germline-specific depletion of DAF-16/FoxO via germline-specific TIR1 expression in fed control animals did not result in changes in brood size, exploratory behavior, or oxidative stress resistance (Fig. 5C). Moreover, both strains recapitulated the fertility defects in F4^L1starved^ animals in response to early life starvation and acute depletion of DAF-16/FoxO in the germline did not affect increased exploration or reduced oxidative stress resistance in F4^L1starved^ + 1^fed^ or F4^L1starved^ + 3^fed^ animals (Fig. 5C and fig. S4G). These results suggest that DAF-16/FoxO function in the germline does not contribute to brood size, exploratory behavior, or oxidative stress resistance under normal conditions. Moreover, they indicate that the observed inter- and transgenerational IIS-dependent phenotypes are not a result of decreased DAF-16/FoxO activity in the germline of F4^L1starved^ + 1^fed^ and F4^L1starved^ + 3^fed^ animals.

Germline-specific depletion of DAF-16/FoxO in the parental generation during acute starvation (Fig. 5D and fig. S4H), recovery (Fig. 5E and fig. S4I), or starvation and recovery continuously (Fig. 5F and fig. S4J) did not improve fertility defects in F4^L1starved^ or affect exploration or oxidative stress resistance in F4^L1starved^ + 1^fed^ and F4^L1starved^ + 3^fed^ animals. Thus, despite the prominent and prolonged nuclear accumulation of DAF-16/FoxO in the germline of L1-starved animals into adulthood, our data indicate that neither acute nor prolonged increased DAF-16/FoxO activity in the germline contribute to the observed IIS-dependent phenotypes in subsequent generations. In conclusion, these findings suggest that IIS does not act directly in the germline to initiate or manifest metabolic programming across generations.

### Starvation-induced increase of DAF-16/FoxO activity in somatic tissues causes metabolic programming across generation

Because *daf-16* does not appear to act in the germline to control metabolic programming, we tested whether *daf-16* is required in the soma and, if so, at what time it is required. Are acute inter- and transgenerational changes in DAF-16/FoxO activity the sole drivers of defects in oxidative stress resistance and exploratory behavior, or does the observed starvation-induced up-regulation of DAF-16/FoxO in the parental generation play a role in initiating mechanisms that ultimately lead to these observed phenotypes? To address these possibilities, we crossed a pansomatic TIR1 transgene (*ieSi57*) into the *daf-16/FoxO::mNG* allele (*ot853*) to deplete DAF-16/FoxO from all somatic tissues except the germline in an auxin-dependent, temporally controlled manner (fig. S5B).

**Fig. 6.**
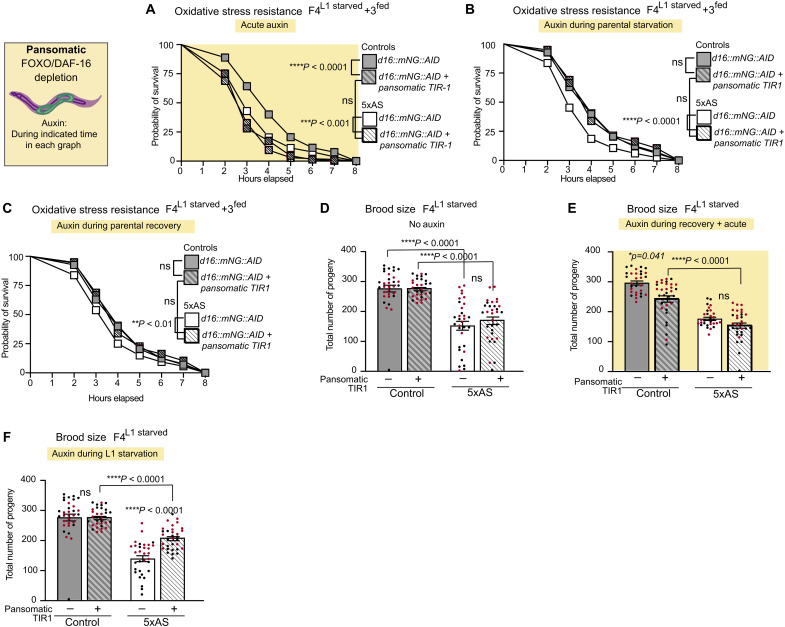
Starvation-induced increase of DAF-16/FoxO activity in somatic tissues causes metabolic programming across generation. In the following experiments, DAF-16/FoxO::mNG::AID was depleted by expressing TIR1 pansomatically and only upon specific addition of auxin during distinct time points as described below: (**A**) Acute pansomatic DAF-16/FoxO depletion decreased oxidative stress resistance in control and F4^L1starved^ + 3^fed^ animals. (**B**) Pansomatic DAF-16/FoxO depletion during the starvation phase in the parental generation only prevented decreased oxidative stress resistance in F4^L1starved^ + 3^fed^ animals. (**C**) Pansomatic DAF-16/FoxO depletion during the recovery phase in the parental generation only prevented decreased oxidative stress resistance in F4^L1starved^ + 3^fed^ animals. Data for all oxidative stress experiments were collected on day 2 of adulthood and are presented as combined data from three independent biological replicates with *n* = 70 to 96 per replicate. Statistical significance was determined by log rank (Mantel-Cox test). (**D**) Pansomatic expression of TIR1 does not affect brood size in the absence of auxin in control or F4^L1starved^ animals. (**E**) Pansomatic DAF-16/FoxO depletion from L1 to adulthood mildly decreased brood size in control animals. Pansomatic DAF-16/FoxO depletion after L1 starvation into adulthood did not suppress the decreased brood size phenotype of F4^L1starved^ animals. (**F**) Pansomatic DAF-16/FoxO depletion exclusively during the L1 starvation phase significantly increased brood size in adult F4^L1starved^ animals. In each panel, all groups were exposed to auxin during the specific time window as indicated in the figure. A yellow background indicates additional acute exposure to auxin during experiment. Data for all brood size experiments were obtained from two independent experiments with *n* = 16 per experiment. Each dot represents one animal. Data are presented as mean ± SEM, and significance was determined by one-way ANOVA with post hoc Tukey. See also fig. S5. ns, not significant.

Acute, pansomatic depletion of DAF-16/FoxO in control fed, as well as F4^L1starved^ + 1^fed^ and F4^L1starved^ + 3^fed^ animals significantly decreased the animals’ oxidative stress resistance compared to their respective controls (Fig. 6A and fig. S5F). Along with our findings from loss-of-function mutants, these results suggest that ancestral starvation results in impaired but not completely abolished DAF-16/FoxO function in somatic tissues, which is responsible for reduced resistance to oxidative stress compared to control animals.

Pansomatic depletion of DAF-16/FoxO during either the starvation phase (Fig. 6B and fig. S5G) or the recovery phase (Fig. 6C and fig. S5H) in the parental generation was sufficient to revert the impaired oxidative stress resistance in subsequent generations. These findings indicate that the prolonged increase in DAF-16/FoxO activity into adulthood as a result of acute up-regulation of DAF-16/FoxO in the soma during starvation is critical in establishing mechanisms that result in IIS-dependent decreased oxidative stress resistance in subsequent generations.

Next, we assessed the role of somatic DAF-16/FoxO signaling in regulating brood size. Pansomatic TIR1 expression in the absence of auxin did not affect brood size in DAF-16::mNG::AID control and F4^L1starved^ animals (Fig. 6D). However, we found that auxin-induced pansomatic DAF-16/FoxO depletion modestly decreased brood size in control animals under normal conditions (Fig. 6E). Moreover, we found that pansomatic DAF-16/FoxO depletion during the starvation phase (Fig. 6F), but not during the recovery phase (Fig. 6E), significantly increased the number of offspring in L1-starved animals, demonstrating that nutritional stress–induced up-regulation of pansomatic DAF-16/FoxO contributes to the observed impaired fertility of L1-starved animals.

We had shown above that constitutive removal of DAF-16/FoxO function increased exploratory behavior in control animals and reverted the difference in exploration between control and ancestrally starved animals (Fig. 3A). However, acute pansomatic depletion of DAF-16/FoxO was insufficient to increase exploration in either control or ancestrally starved animals (fig. S5I). In addition, pansomatic DAF-16/FoxO depletion during either the starvation phase (fig. S5J) or the recovery phase (fig. S5K) in the parental generation also did not prevent increased exploration in subsequent generations. Thus, postembryonic, conditional depletion of DAF-16/FoxO from either the germline or all somatic tissues using the AID/TIR1 system is not sufficient to acutely impact exploratory behavior in control or ancestrally starved animals (please also see Discussion). Together, our findings point to distinct roles of DAF-16/FoxO activity in somatic tissues during specific developmental time windows in initiating metabolic programming, resulting in IIS-dependent phenotypes across generations.

### Starvation-induced changes in neuronal and intestinal DAF-16/FoxO signaling contribute to parental sterility and descendant’s impaired stress resistance

Last, we aimed to further narrow down the somatic tissue(s) in which changes in DAF-16/FoxO action are required to initiate and manifest metabolic programming. We determined the effects of DAF-16/FoxO depletion specifically from the nervous system and the intestine (fig. S5, C and D), because (i) both tissues are important regulators of metabolism and (ii) our transcriptome analysis between fed control versus F4^L1starved^ + 3^fed^ animals pointed to an enrichment of genes specifically expressed in these tissues (Fig. 2C). Here, we crossed animals expressing the *daf-16/FoxO::mNG* allele to animals expressing TIR1 under the control of either a panneuronal (*rgef-1*; *reSi7*) or intestine-specific (*ges-1*; *ieSi61*) promoter and exposed the animals to auxin during distinct developmental and generational time points as described above.

**Fig. 7.**
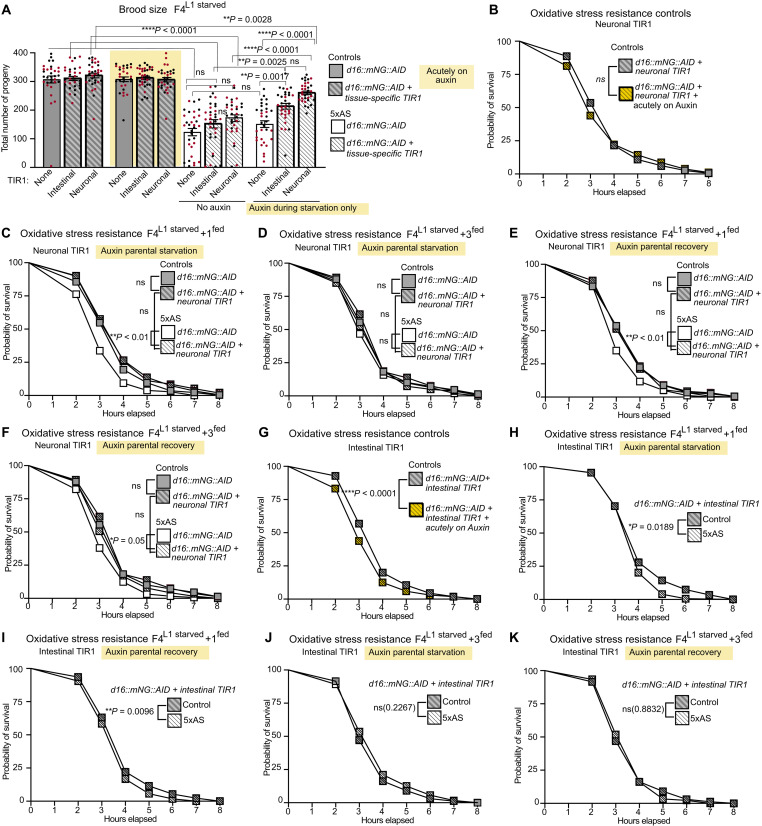
Starvation-induced changes in intestinal and neuronal DAF-16/FoxO signaling contribute to parental sterility and descendant’s impaired stress resistance. DAF-16/FoxO::mNG::AID was depleted by expressing TIR1 under the control of a panneuronal or intestine-specific promoter upon specific addition of auxin during distinct time points: (**A**) Intestine- and neuron-specific DAF-16/FoxO depletion exclusively during L1 starvation improved fertility defects of F4^L1starved^ animals. Brood size was determined from two independent biological replicates with *n* = 16 per replicate. Data are presented as mean ± SEM. Each dot represents one animal. Statistical significance was determined by one-way ANOVA with post hoc Tukey. (**B**) Acute depletion of DAF-16/FoxO from the nervous system did not affect oxidative stress resistance to paraquat in control animals. (**C**) Neuronal depletion of DAF-16/FoxO exclusively during L1 starvation reverted decreased oxidative stress resistance in F4^L1starved^ + 1^fed^ and (**D**) F4^L1starved^ + 3^fed^ animals. The no-TIR1 F4^L1starved^ + 3^fed^ animals did not display the usually observed decreased oxidative stress resistance due to one of three replicates performed. (**E**) Neuronal depletion of DAF-16/FoxO during the recovery phase in the parental generation only reverted decreased oxidative stress resistance in F4^L1starved^ + 1^fed^ and (**F**) F4^L1starved^ + 3^fed^ animals. (**G**) Acute DAF-16/FoxO depletion from intestine decreased oxidative stress resistance in control animals. (**H**) Intestinal DAF-16/FoxO depletion during L1 starvation or (**I**) the recovery phase only in the parental generation is not sufficient to revert decreased intergenerational oxidative stress resistance in F4^L1starved^ + 1^fed^ animals. (**J**) Transgenerational decrease in oxidative stress resistance is reverted upon intestinal DAF-16/FoxO depletion during L1 starvation or (**K**) recovery period of the parental generation. In each panel, groups were exposed to auxin during the specific time window as indicated. A yellow background indicates additional acute exposure to auxin during experiment. Data for oxidative stress experiments were collected on day 2 of adulthood and are presented as combined data from two to three independent biological replicates with *n* = 80 to 103 per replicate. Statistical significance was determined by log rank (Mantel-Cox test). ns, not significant.

Tissue-specific DAF-16/FoxO depletion during acute starvation from either the nervous system or intestine (fig. S5, C and D) significantly improved fertility defects observed in F4^L1starved^ animals (Fig. 7A). Thus, IIS-dependent signals generated in response to nutritional stress during early life in both the intestine and nervous system contribute to fertility defects in adulthood.

Oxidative stress resistance was not affected by acute removal of neuronal DAF-16/FoxO function in control animals (Fig. 7B). However, neuronal DAF-16/FoxO depletion during either starvation (Fig. 7, C and D) or recovery (Fig. 7, E and F) in the parental generation was sufficient to improve oxidative stress resistance in F4^L1starved^ + 1^fed^ and F4^L1starved^ + 3^fed^ animals. These results suggest that although neuronal DAF-16/FoxO might play a negligible role in the animals’ acute response to oxidative stress (Fig. 7B), starvation-induced up-regulation of neuronal DAF-16/FoxO is critical for initiating mechanisms that determine oxidative stress resistance in subsequent generations.

Interpretation of results obtained for oxidative stress resistance in response to intestinal DAF-16/FoxO depletion proved to be more challenging. The intestinal TIR1-expressing control strain displayed an increased oxidative stress resistance compared to the no TIR1 control strain even in the absence of auxin (fig. S6A). We therefore only compared the effects of DAF-16/FoxO depletion between intestinal TIR1-expressing animals. Acute depletion of DAF-16/FoxO in the intestine slightly decreased oxidative stress resistance in control animals (Fig. 7G), demonstrating that DAF-16/FoxO signaling in the intestine is necessary to elicit a complete oxidative stress response. Moreover, although intestinal DAF-16/FoxO depletion during either the starvation (Fig. 7H) or recovery phase (Fig. 7I) in the parental generation did not fully revert the intergenerational decreased oxidative stress resistance in F4^L1starved^ + 1^fed^ animals, decreased oxidative stress resistance was no longer seen transgenerationally in F4^L1starved^ + 3^fed^ compared to control animals (Fig. 7, J and K). These results could point to distinct mechanisms responsible for mediating intergenerational versus transgenerational metabolic programming phenotypes. Alternatively, it is possible that the perseverance of metabolic programming phenotypes across generations is, in part, determined by the relative amount of signal generated in response to nutritional stressors across different tissues in the ancestral generation that experienced early life starvation.

## DISCUSSION

**Fig. 8.**
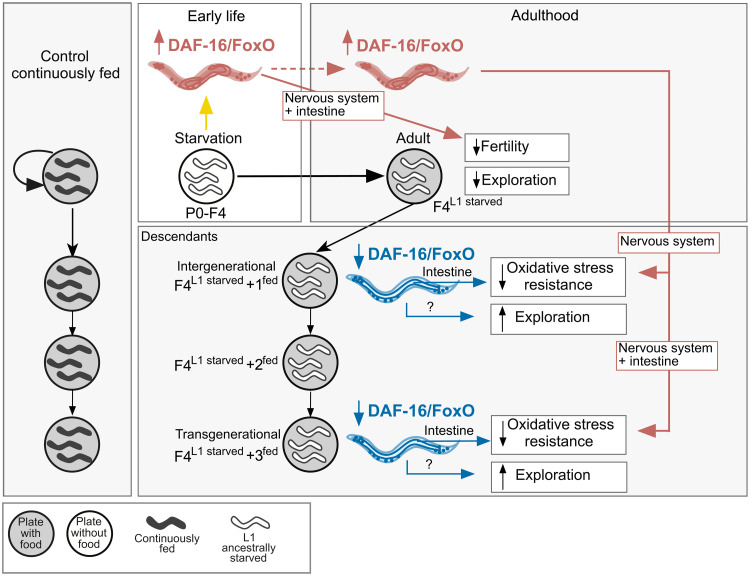
Proposed model how starvation-induced acute and transgenerational changes in somatic IIS drive metabolic programming across generations. Acute starvation during early life increases DAF-16/FoxO in somatic tissues, which persists into adulthood. Increased DAF-16/FoxO activity in the nervous system and intestine during starvation contribute to decreased fertility in adults. Moreover, the prolonged increased DAF-16/FoxO activity into adulthood is critical in initiating mechanisms that lead to decreased oxidative stress resistance inter- and transgenerationally. Early life starvation–induced increase in DAF-16/FoxO in the nervous system and intestine is involved in initiating mechanisms resulting in transgenerational effects on oxidative stress resistance. Inter- and transgenerational decrease in DAF-16/FoxO function in the intestine is, at least in part, responsible for manifesting decreased oxidative stress resistance in each generation. Although our data show that inter- and transgenerational increases in exploration depend on IIS, at this point we do not know when and where IIS acts to mediate this phenotype across generations. Gray circle, food; white circle, no food; dark gray worms, continuously fed controls; white worms, animals that experienced L1 starvation in parental generation. Red color indicates increased activity, whereas blue color indicates decreased activity.

By using a recurrent starvation paradigm in *C. elegans*, we provide direct evidence that DAF-16/FoxO, the main downstream target of the IIS pathway, plays critical and distinct roles in metabolic programming (Fig. 8).

A critical role for altered insulin signaling in metabolic programming has been suggested as early as the 1950s, when first correlations between maternal glucose levels, fetal hyperinsulinemia, and adverse health outcomes in the offspring were made (*73*). Since then, an increasing amount of human epidemiological and animal studies have provided additional evidence that nutritional and metabolic stressors affect insulin levels, as well as insulin sensitivity, ultimately affecting the activity of the insulin signaling pathway and its downstream targets not only in the parental but also in subsequent generations (*74*–*76*). However, only very few studies were able to establish a causal link between altered insulin signaling and molecular, cellular, and metabolic phenotypes intra- or intergenerationally (*30*, *41*, *77*). To our knowledge, our study is the first to provide mechanistic evidence that nutrition-induced changes in somatic insulin signaling are directly responsible for molecular, physiological, and behavioral impairments transgenerationally.

Despite the relative simplicity of *C. elegans*, the striking similarities in starvation-induced phenotypes we observe in our study and metabolic programming phenotypes described in rodents and humans underscore the relevance of our findings in deciphering fundamental mechanisms underlying adverse health outcomes across generations. For example, malnutrition and impaired metabolic health significantly impair both male and female fertility in humans (*78*). We demonstrate that such defects in fertility are partially mediated by acute diet-induced changes in IIS, specifically via DAF-16/FoxO signaling in somatic tissues, including the intestine and nervous system. Moreover, maternal obesity and high-fat diet (HFD) consumption correlate with elevated markers of oxidative stress in their offspring in rodents (*79*, *80*). We found that early life starvation impairs oxidative stress resistance not only intergenerationally but also transgenerationally. In addition, we identified that the starvation-induced changes in somatic DAF-16/FoxO activity, which continue throughout the animals’ lifetime at least until early adulthood, play an important role in establishing mechanisms that lead to these physiological changes across generations. A recent study determined that maternal exposure to osmotic stress improved the offspring’s ability to survive under similar osmotic conditions and that this intergenerational adaptation of osmotic stress resistance was dependent on maternal DAF-16/FoxO signaling in the germline (*81*). Thus, DAF-16/FoxO seems to affect physiology and behavior across generations in a stress-dependent manner and via tissue-specific mechanisms. Overall, our findings further highlight the importance of well-balanced insulin levels throughout the lifetime of an organism to prevent a multitude of adverse health outcomes in subsequent generations.

The increased exploratory phenotype we observed in ancestrally starved animals under fed conditions resembles the recently described increased novelty-induced hyperlocomotion in male offspring of postnatally HFD-fed mothers in mice (*82*), pointing to a potential evolutionary conservation of this phenomenology. We demonstrate that changes in exploratory behavior depend on IIS. However, we were unable to identify the timing and cellular focus of aberrant IIS in mediating this behavior using the conditional AID/TIR1 system. It is possible that our approach did not sufficiently remove DAF-16/FoxO to fully recapitulate the loss-of-function phenotype or that DAF-16/FoxO acts in both the germline and somatic tissues to regulate behavior on food. Alternatively, increased exploratory behavior could have been mediated by nutrition-induced changes in IIS during embryonic development. In our hands, auxin exposure did not deplete DAF-16/FoxO in embryos expressing TIR1 (fig. S5E); this may be particularly important, because the AIY interneuron, a neuron previously shown to be critically involved in exploratory behavior (*83*, *84*), requires DAF-16/FoxO for proper axon outgrowth during development (*85*).

One of the major questions that remains is how nutrition-induced changes in somatic IIS in the parental generation lead to altered IIS-dependent molecular, behavioral, and physiological phenotypes across generations. As indicated earlier, developmental processes are highly sensitive to changes in nutritional, hormonal, and metabolic environment. Fetal hyperinsulinemia in the brain is associated with decreased expression of proliferative genes in the hypothalamus, potentially contributing to observed changes in neuronal cell number (*76*). Moreover, aberrant insulin signaling as a result of maternal HFD feeding contributes to impaired axon innervation of hypothalamic pro-opiomelanocortin neurons to a specific downstream intrahypothalamic target area, which was associated with impaired glucose tolerance in the offspring in mice (*30*). Thus, such direct developmental defects mediated by changes in signaling pathways regulating metabolism can predispose offspring of malnourished parents for an impaired metabolic health throughout lifetime. These metabolic impairments in the immediate offspring could be sufficient to again create an aberrant metabolic environment during development of the next generation, thereby creating a cascade of metabolic programming across generations (*86*).

In addition to such direct developmental defects from imbalanced metabolic signaling events, there is growing evidence that diet- and metabolism-mediated changes in the epigenome particularly in the germline contribute to metabolic programming across generations. How could germline-generated signals play any role in inter- and transgenerational phenotype development in our paradigm in which starvation occurs during the L1 stage, during which *C. elegans* only has two germline precursor cells? Signals generated as a response to acute starvation would be diluted during subsequent proliferation and meiotic development until the fully mature germline is present in adult animals. However, our findings that early life starvation leads to drastic molecular changes that last into adulthood highlight the prolonged exposure and, thus, vulnerability of the germline to metabolic changes that go beyond the time window of acute stress exposure. This “metabolic rewiring” of the exposed animal, in particular somatic changes in IIS, could lead to the generation of “heritable” changes in the germline, including epigenetic signals, at any time point and thereby contribute to phenotype development in subsequent generations. Over the past decade, an increasing amount of human and animal studies have found that parental diet and metabolism affect patterns of DNA methylation, histone modifications, and levels of noncoding RNAs not only in the parents but also in their offspring (*4*, *87*, *88*). A large subset of genes encoding for critical factors involved in the biogenesis and function of small RNAs are class 2 downstream targets of IIS (i.e., *dcr-1*, *drh-3*, *rde-4*, *rrf-1*, *hrde-1*, *mut-14*, *mut-16*, *nrde-2*, *nrde-4*, and *sago-2*) (*65*), and we found a significant down-regulation of some of these genes in adult animals that experienced starvation during early life compared to consistently fed control animals (fig. S7). A previous study suggested that L1 starvation in *C. elegans* directly influences the small RNA pool of the starved animal throughout its lifetime and that some of these epigenetic changes are inherited transgenerationally (*43*). Another study was able to demonstrate that neuronally generated RDE-4–dependent small RNAs are able to modify gene expression transgenerationally (*89*), including expression of *saeg-2*, which had been implicated in the regulation of foraging behavior (*90*). Last, metabolism pathways also generate and depend on several metabolites (e.g., acetyl–coenzyme A, *S*-adenosyl-l-methionine, adenosine 5′-triphosphate, and nicotinamide adenine dinucleotide) that are necessary for regulatory modifications of chromatins (*91*). Thus, it is conceivable that there is a dynamic interplay between changes in metabolism and the biogenesis of epigenetic factors that could in turn contribute to metabolic programming across generations. However, whether the observed changes in epigenetic signals are causative to or a result of changes in metabolism across generations and which role they play in our paradigm of metabolic programming still remains to be deciphered.

The range of fertility defects seen in L1-starved adult animals could also point to nutritional stress–induced selection for animals or a subset of germ cells with distinct intrinsic features that provide advantages to survive and recover from such adverse conditions. These intrinsic features could in turn be inherited and, thus, be responsible for changes in physiology and behavior in subsequent generations. For example, while fertile F4^L1starved^ animals responded with a strong up-regulation of DAF-16/FoxO target genes compared to control fed animals, this response was blunted compared to the even stronger up-regulation seen in sterile F4^L1starved^ animals (fig. S3A and data S12). If this blunted response was due to heritable, intrinsic changes in fertile F4^L1starved^ animals, the following generation would then be enriched for animals with relatively reduced activity of DAF-16/FoxO, which was one of the key findings from our study. The nature of such intrinsic features could include any heritable signals, such as DNA mutations, epigenetic signals, or mitochondria. However, to date, we do not know why some animals recover from L1 starvation better than others, and do not have any evidence in support of or against such a selection-driven mechanism underlying metabolic programming.

None of the hypotheses discussed above in regard to how nutrition-induced changes in IIS might mediate metabolic programming are mutually exclusive. Although a large number of studies in the field of metabolic programming has focused on identifying changes in the epigenome that could underlie observed inter- and transgenerational phenotypes, the current lack of causative evidence between phenotypes and epigenetic changes demonstrates the complexity of, and highlight the necessity for engaging in in-depth, detailed dissections of mechanisms underlying intra-, inter-, and transgenerational metabolic programming. Thus, future studies are required to decipher the potential interplay and dependency between IIS, metabolic changes, epigenetic signals, and developmental processes and to dissect their organ-specific roles that mediate their relative contribution to metabolic programming across generations.

## MATERIALS AND METHODS

### *C. elegans* strains and handling

Worms were grown at 20°C on nematode growth medium (NGM) plates seeded with *Escherichia coli* (OP50) bacteria as food source unless otherwise mentioned. Worms were maintained according to standard protocols. The wild-type strain used was the Bristol variety, strain N2. A complete list of strains and transgenes used in this study is provided in Table 1. All strains used for our recurrent starvation paradigm were maintained for at least five generations under well-fed and clean conditions before paradigm was started.

**Table 1.** List of *C. elegans* strains used in this study.

Strain	Source	Identifier
N2	Caenorhabditis Genetics Center (CGC)	Wormbase: N2; Wormbase: WBStrain 00000001
*daf-16(mu86)*	(*51*)	CF1038
*daf-2(e1370)*	(*61*)	OH8521 (outcrossed to laboratory N2 4×)
*ot853*	(*48*)	OH14125
*ieSi38*	(*72*)	CA1199
*ieSi57*	(*72*)	CA1200
*ieSi61*	(*72*)	CA1209
*reSi7*	(*103*)	DV3805
*ot853; ieSi38*	This study	OH17836
*ot853; ieSi57*	(*48*)	OH14345
*ot853; ieSi61*	(*48*)	OH17003
*ot853; reSi7*	This study	OH17835

### Recurrent L1 starvation paradigm

Eggs were harvested from well-fed and clean hermaphrodites at day 1 of adulthood by egg prep: Adult hermaphrodites were exposed to bleach solution [0.5 M NaOH in 1.5% bleach (Clorox) in H_2_O] for 5 min under vigorous shaking. Bleached eggs were washed four times with M9. Eggs were plated at around 500 eggs per plate on 6-cm NGM plates with food (control) or without food (starved L1). Starved animals were starved on the same plate for a period of 6 days, after which they were fed with OP50. Three days after reexposure to food, L1-starved, adult hermaphrodites were exposed to bleach solution and harvested eggs were again plated on NGM plates without food. This form of starvation was repeated for five consecutive generations total (P0-F4^L1starved^). After five generations of L1 starvation, eggs from day 1 adult hermaphrodites were harvested by egg prep and plated on NGM plates with food (F4^L1starved^ + 1^fed^). Once F4^L1starved^ + 1^fed^ animals reached day 1 of adulthood, eggs were harvested by egg prep and plated on NGM plates with food. Last, eggs from F4^L1starved^ + 2^fed^ animals were harvested and plated on NGM plates with or without food, depending on the experiment (F4^L1starved^ + 3^fed^). Control animals were maintained under well-fed conditions at all times, and at day 1 of adulthood, eggs were harvested by egg prep and plated on NGM plates with food until ancestrally starved animals reached F4^L1starved^ + 2^fed^. For final experiments, eggs from control and ancestrally starved animals were harvested at the same time. For each generation, eggs were plated on at least eight plates per condition. Each plate on which animals were accidentally exposed to starvation outside of planned L1 starvation, or any plates with contaminated were discarded.

### Single-worm tracking analysis

Automated single-worm tracking analysis was performed using the WormTracker 2.0 system (*56*) on control and F4^L1starved^ + 3^fed^ animals. For tracking on food, 12 μl of OP50 was added to the center of a 6-cm NGM plate and allowed to dry for 30 min. One L4 animal was transferred from a well-fed plate onto the food patch and was immediately tracked for 5 min. The same animal was tracked a second time for 5 min, 30 min after initial transfer. For tracking off food, one L4 animal was transferred from a well-fed plate onto an NGM plate without food, let move around for 1 min (to remove most of the food attached to body), and then transferred onto 5-cm tracking plate without food and tracked for 5 min. The same animal was tracked a second time for 5 min, 30 min after initial transfer on the same plate without food. Tracking of control and ancestrally starved animals was performed on the same day with *n* = 10 animals per group per day and condition. Order of tracked animals was randomized between groups each day. Once animals moved to the edge of the plate, the tracker automatically stopped tracking and animals had to be excluded from analysis. We performed two independent biological replicates with *n* = 10 animals as pilot experiments to identify potentially altered behavior on and off food using the accompanying tracking analysis software. For subsequent, additional analyses of three independent biological replicates with *n* = 10 per group and replicate on food and *n* = 15 per group and replicate off food, we only focused on features previously identified in pilot experiments. Only path range was consistently altered between control and ancestrally starved animals under fed conditions in pilot and final experiments. Statistical analysis [one-way analysis of variance (ANOVA) with post hoc Tukey] was performed on combined data from three independent biological replicates.

### Exploratory behavior

To measure exploratory behavior, we used an exploratory assay as previously described (*84*). Briefly, 35-mm NGM plates were fully covered with OP50 and allowed to dry for 2 to 3 days. One young L4 animal was transferred to each plate. After 16 hours, the plates were superimposed on a grid containing 3.5-mm squares, and the number of squares entered (maximum of 88) by each worm was manually counted. Ancestrally starved animals were always compared with control animals assayed in parallel. For each assay, 15 to 20 animals per group and replicate were analyzed. Statistical significance was determined using unpaired Student’s *t* test or, when more than two groups were analyzed, one-way ANOVA with post hoc Tukey’s.

### Life span assay

To determine differences in life span in F4^L1starved^ + 3^fed^ animals, 10 × 10 L4 animals were picked onto 6-cm NGM plates with OP50. The animals were transferred every other day until day 10 of adulthood. The animals were scored [dead/alive/censored(=bagged/missing/plate contaminated)] every other day until all animals were deceased. Animals were censored when lost or bagging occurred. The experiment was repeated for three independent biological replicates. To determine differences in life span in P0^L1starved^ + 3^fed^ animals, experiments were performed as described above, except that the first biological replicate was started with 5 × 10 L4 animals, and the second biological replicate was started with 10 × 10 L4 animals. Statistical significance was determined with Gehan-Breslow-Wilcoxon test in GraphPad Prism. Biological replicates are displayed in one graph.

### Oxidative stress assay

Oxidative stress resistance was measured at day 2 of adulthood using the herbicide paraquat (methyl viologen dichloride hydrate, Sigma-Aldrich 856177) as previously described (*92*) with slight modifications. For each group, 10 to 12 well-fed animals were transferred per well to a total of 8 or 12 wells containing 70 μl of paraquat solution in M9 [100 mM for experiments using only N2 wild-type animals, 80 mM for experiments using N2 and *daf-16(mu86)* animals, as well as for all AID/TIR1 experiments, and 120 mM using *daf-2(e1370)* animals]. Death of each animal was scored every hour over 8 hours. Each condition was tested at least two independent times. Data from independent replicates were combined for final figure and statistical analysis (log-rank Mantel-Cox test).

### Measurement of intracellular ROS

ROS levels were determined in L1-fed animals using 2′,7′-dichlorodihydrofluorescein diacetate (H_2_DCFDA; Sigma-Aldrich, D6883) as previously described (*93*). Briefly, eggs from control and ancestrally starved (F4^L1starved^ and F4^L1starved + 2fed^) were harvested by egg prep (see starvation paradigm) and placed on NGM plates with or without OP50. Twelve hours after egg prep, worms were collected, washed five times with M9, and diluted in M9 to reach a final concentration of 50 animals/10 μl. Ten microliters of worm suspension was added to 40 μl of M9 in 16 wells per group of a 96-well plate. Fifty microliters of 50 μM H_2_DCFDA was added per well, and fluorescence intensity was measured at an excitation wavelength of 490 nm and an emission wavelength of 530 nm after 0, 60, 120, 180, and 240 min. The 96-well plate was protected from light throughout the experiment. Blank-subtracted raw data of ancestrally starved were normalized to those in control animals at each time point, and the average difference between 60, 120, 180, and 240 min was calculated and used for statistical analysis (unpaired Student’s *t* test). After 240 min, each well was inspected and values from wells that contained any kind of debris were excluded from analysis.

### Transcriptome analysis

Transcriptome analysis of ancestrally starved animals (F4^L1starved^ + 3^fed^) was performed on ~10,000 L1 animals per replicate on three independent replicates. L1 control and ancestrally starved animals were collected from NGM plates with food 14 hours and without food 18 hours after egg prep. Animals were washed 4× with M9 and centrifuged at 500*g* for 2 min. After the last wash, 200 μl of TRIzol (Invitrogen) was added to each worm pellet. Worm pellets were homogenized for 30 s using an automated pestle and then placed on dry ice for 5 min. After a total of four rounds of homogenization and freezing, homogenized worms were stored at −80°C until further use. For RNA isolation, additional 600 μl of TRIzol (Thermo Fisher Scientific, 15596026) was added to homogenized worms and warmed up to room temperature. After the addition of 160 μl of chloroform and 5 min of vortexing, tubes were centrifuged for 15 min at 4°C and maximum speed. The upper layer was transferred to a fresh tube, mixed 1:1 with 70% EtOH, and then transferred to RNeasy MinElute spin columns (Qiagen). RNA extraction was performed according to RNeasy MinElute protocol. High-quality RNA was used for library preparation (NuGEN Ovation Universal RNA-Seq) with custom InDA-C primers for *C. elegans*. Libraries were sequenced using NextSeq 500 sequencing machines with 75–base pair single-end reads (Illumina). After the initial quality check, the reads were mapped to WS220 using the Subread package (*94*) and assigned to genes using featurecounts (*95*). Differential gene expression analysis was performed using DESeq2 (*96*). Heatmaps and volcano plots were generated using R packages heatmap3 and ggplot2 (*97*, *98*). Downstream target genes of signaling pathways regulating metabolism were identified from previously published studies (*65*–*68*). Enrichment analyses were performed with tools available on wormbase.org (*99*, *100*).

Samples for transcriptome analysis of control, fertile F4^L1starved^ and sterile F4^L1starved^ were collected as follows: For F4^L1starved^ animals, around 1000 L4 animals (at this stage, adult fertility is not obvious) were transferred to a fresh NGM plate with food. After 24 hours, 100 adult hermaphrodites with >5 eggs (fertile F4^L1starved^ sample) and 100 adult hermaphrodites without eggs (sterile F4^L1starved^ sample) were collected. Simultaneously, 100 day 1 adult control animals with >5 eggs were collected. RNA isolation, library preparation, sequencing, and analysis were performed as described above. Normalized counts from all samples and for all transcripts can be found in data S1 and S6.

### Brood size quantification

For determination of brood size, 16 animals per group for each replicate were singled at the L4 larval stage and transferred daily until egg-laying ceased. Viable progeny was counted 72 hours after eggs were laid. Ancestrally starved animals were always compared with control animals assayed in parallel. Data were obtained from two independent biological replicates. Statistical significance was determined using Mann-Whitney *U* test or, when more than two groups were analyzed, one-way ANOVA with post hoc Tukey’s.

### Imaging/microscopy

Worms were anesthetized using 50 mM sodium azide and mounted on 5% agarose on glass slides. All images were acquired using a Zeiss confocal microscope (LSM880). Image reconstructions were performed using Zen software tools. Maximum intensity projections of representative images are shown. Scale bars are 15 μm.

### Quantification of nuclear accumulation of DAF-16::mNG in germline

To determine subcellular localization of DAF-16 in the germline of early life starved animals, control and fertile as well as sterile F4^L1starved^ animals at day 1 of adulthood were imaged at 40× using a Zeiss confocal microscope (LSM880) as described above under well-fed conditions. All images from one slide were acquired within a 10-min period, although we did not notice a change in DAF-16 localization in the germline even after longer periods of exposure to sodium azide. Images were scored as “cytoplasmic” or “nuclear” depending on localization of fluorescent signal in the mitotic germline of each animal. Data were obtained from four independent biological replicates with *n* = 10 per replicate. Moreover, acutely starved (>2 hours) control animals at day 1 of adulthood were imaged to demonstrate unique and distinct nuclear accumulation of DAF-16::mNG in the germline of early life starved but currently fed F4^L1starved^ animals compared to acutely starved, control animals (fig. S4B).

### Spatiotemporal DAF-16/FoxO depletion using AID/TIR1 system

For spatiotemporal depletion of DAF-16/FoxO, 6-cm NGM plates containing 4 mM water-soluble auxin solution (naphthaleneacetic acid, K-NAA; Phytotechlab N610) were prepared at least 2 days, but a maximum of 4 days before beginning of each experiment. All plates from auxin experiments were kept in the dark. For DAF-16/FoxO depletion during acute L1 starvation, eggs were directly plated onto NGM plates containing 4 mM auxin without food. The animals were maintained on the same plate until the end of starvation, after which they were washed 3× in M9 and transferred to NGM plates with OP50 bacteria. For DAF-16/FoxO depletion during the recovery phase, L1-starved animals (starvation on NGM plate without food) were transferred onto NGM plates containing 4 mM auxin and OP50 bacteria. We used water-soluble auxin (*101*) to avoid side effects from ethanol, which can act as a carbon source during starvation (*102*). For continuous DAF-16/FoxO depletion, eggs were plated onto NGM plates with auxin without food for the duration of starvation and subsequently transferred to NGM plates with 4 mM auxin and OP50 bacteria. The conditional *daf-16* allele (*daf-16/FoxO::mNG; ot853*) (*48*) was crossed with germline-specific (*ieSi38*), pansomatic (*ieSi57*), intestinal (*ieSi61*) (*72*), or panneuronal (*reSi7*) (*103*) TIR1-expressing transgenic lines. We observed robust and specific green fluorescent protein depletion upon auxin exposure (fig. S5) for all TIR1-expressing strains. As controls, animals expressing the *daf-16/FoxO::mNG* allele but without TIR1 expression were exposed to auxin during the same time period and were included for comparison. For each biological replicate and all experiments, all groups of one TIR1 line (and respective no TIR1 controls) were assayed at the same time. To increase clarity in presentation of data, not all groups that were assayed at the same time are presented in one graph, but rather separated according to time of auxin exposure.

### Statistical analyses

All statistical analyses were performed in GraphPad Prism, except for differential gene expression analysis and enrichment analyses, which were performed in RStudio. Statistical analyses performed for each experiment are described in the figure legends.

## Notes

View/request a protocol for this paper from *Bio-protocol*.
