# Supplementary material for: Starvation-induced changes in somatic insulin/IGF-1R signaling drive metabolic programming across generations
Source: Sci Adv. 2023 Apr 7;9(14):eade1817. doi: 10.1126/sciadv.ade1817 (PMC10081852; doi:10.1126/sciadv.ade1817)
Supplement: Supplementary file 1 — Figs. S1 to S7 Legends for data S1 to S12 [file sciadv.ade1817_sm.pdf]

Science Advances
AAAS

Supplementary Materials for

Starvation-induced changes in somatic insulin/IGF-1R signaling drive metabolic programming across generations

Merly C. Vogt *et al.*

Corresponding author: Merly C. Vogt, merly.vogt@helmholtz-munich.de

Sci. Adv. **9**, eade1817 (2023)
DOI: 10.1126/sciadv.ade1817

The PDF file includes:

Figs. S1 to S7
Legends for data S1 to S12

Other Supplementary Material for this manuscript includes the following:

Data S1 to S12

Supplementary Figure 1

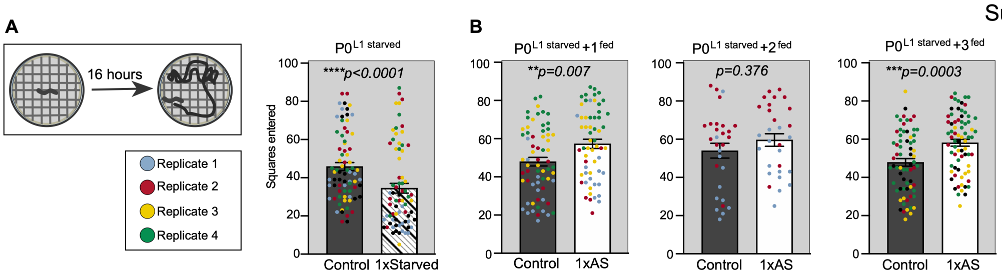

Fig. S1. Inter- and transgenerational increase in exploration can be observed after one round of ancestral starvation, related to Fig. 1

(A) Schematic overview of exploratory behavior assay. **(B)** Exploratory behavior was assessed over a 16h period starting with animals at the young L4 stage. Data was obtained from 2-4 independent biological replicates (indicated by different colors of each data point) with n=15-20 animals/replicate. Data is presented as mean +/-SEM. Significance was determined by Mann Whitney U test.

Supplementary Figure 2

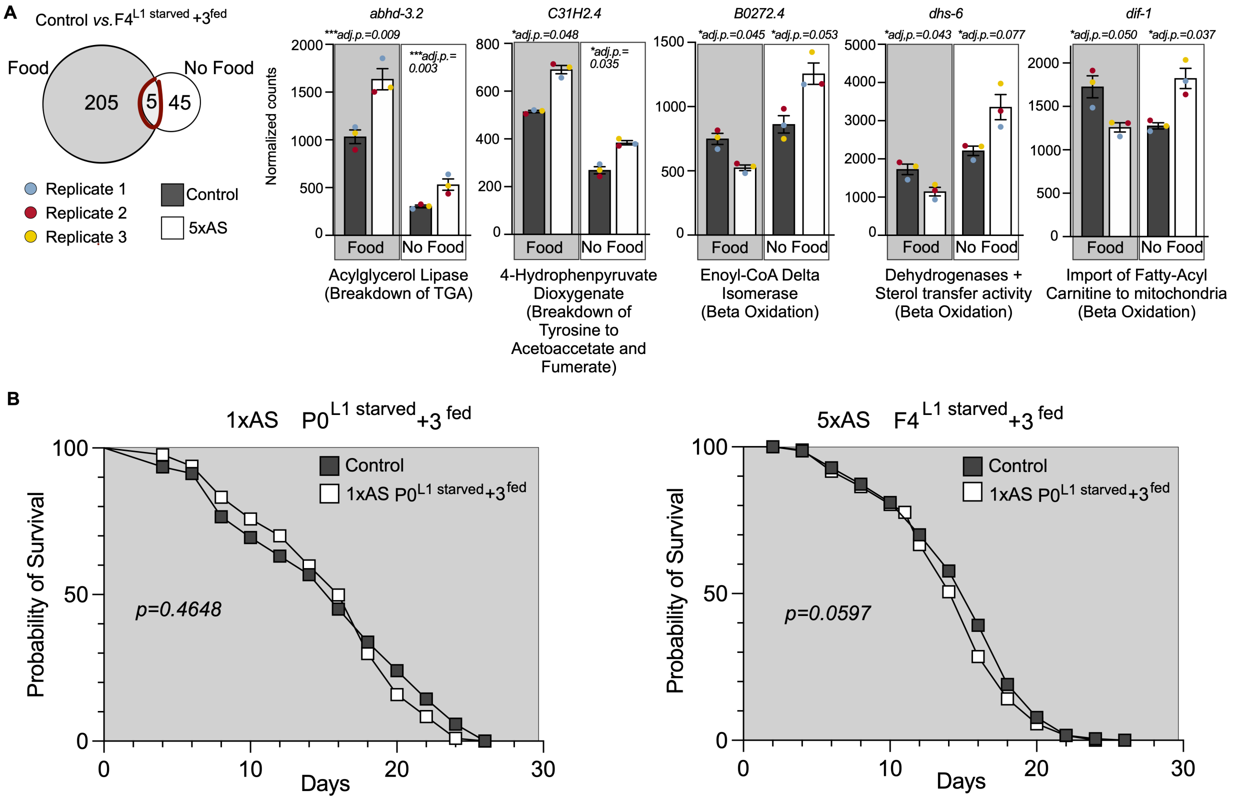

Fig. S2, Early life starvation results in differential expression of genes involved in lipid metabolism transgenerationally, but does not increase lifespan, related to Fig. 2

(A) Transcriptome analysis was performed across 3 independent biological replicates with ~10.000 L1 animals/replicate. Samples were collected under fed and starved conditions. Adjusted p-values were determined by Wald test with subsequent Benjamini and Hochberg correction using the DESeq2 package *(97)*. **(B)** In our hands, early life starvation does not increase lifespan transgenerationally in either $P0^{L1starved}+3^{fed}$ (n control= 139; n 1xAS=130; data combined from two independent biological replicates) or $F4^{L1starved}+3^{fed}$ (n Control=381; n 5xAS=399; data combined from four independent biological replicates) as previously reported *(42, 43)*.

Supplementary Figure 3

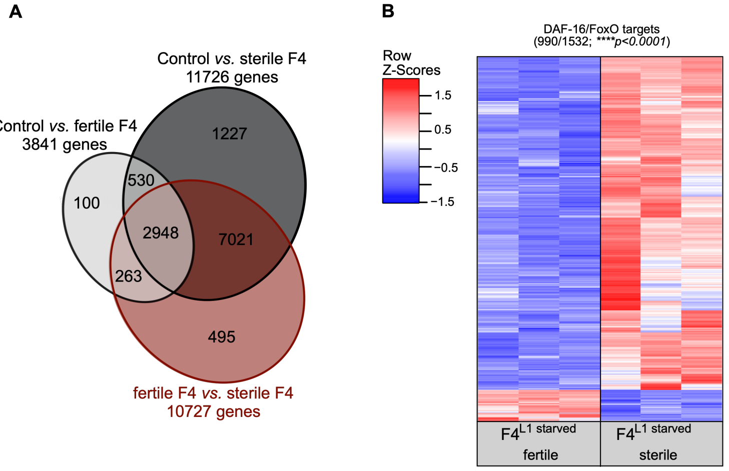

Fig. S3, L1 starvation affects fertility and transcriptome of individuals within a population to different extents, related to Fig. 4

(A) Transcriptome analysis was performed across 3 independent biological replicates with ~100 manually picked animals/group at day 1 of adulthood under fed conditions. Animals were separated into fertile and sterile $F4^{L1starved}$. Differential gene expression between control, fertile and sterile $F4^{L1starved}$ animals and overlap between groups is displayed as Venn-Diagram (not drawn to scale). **(B)** Downstream DAF-16/FoxO target genes that showed an overall relative upregulation in fertile $F4^{L1starved}$ animals compared to control animals (Fig. 3B), showed an even further upregulation in sterile $F4^{L1starved}$ compared to fertile $F4^{L1starved}$ animals. Values in heatmap were z-scored normalized and plotted using heatmap3 in RStudio. Each row represents a single gene and each column represents a single RNA-seq replicate. Blue=relative downregulation, red=relative upregulation in sterile $F4^{L1starved}$ animals. Adjusted p-values were determined by Wald test with subsequent Benjamini and Hochberg correction using the DESeq2 package *(97)*. Significant enrichment was determined by hypergeometric distribution.

Supplementary Figure 4

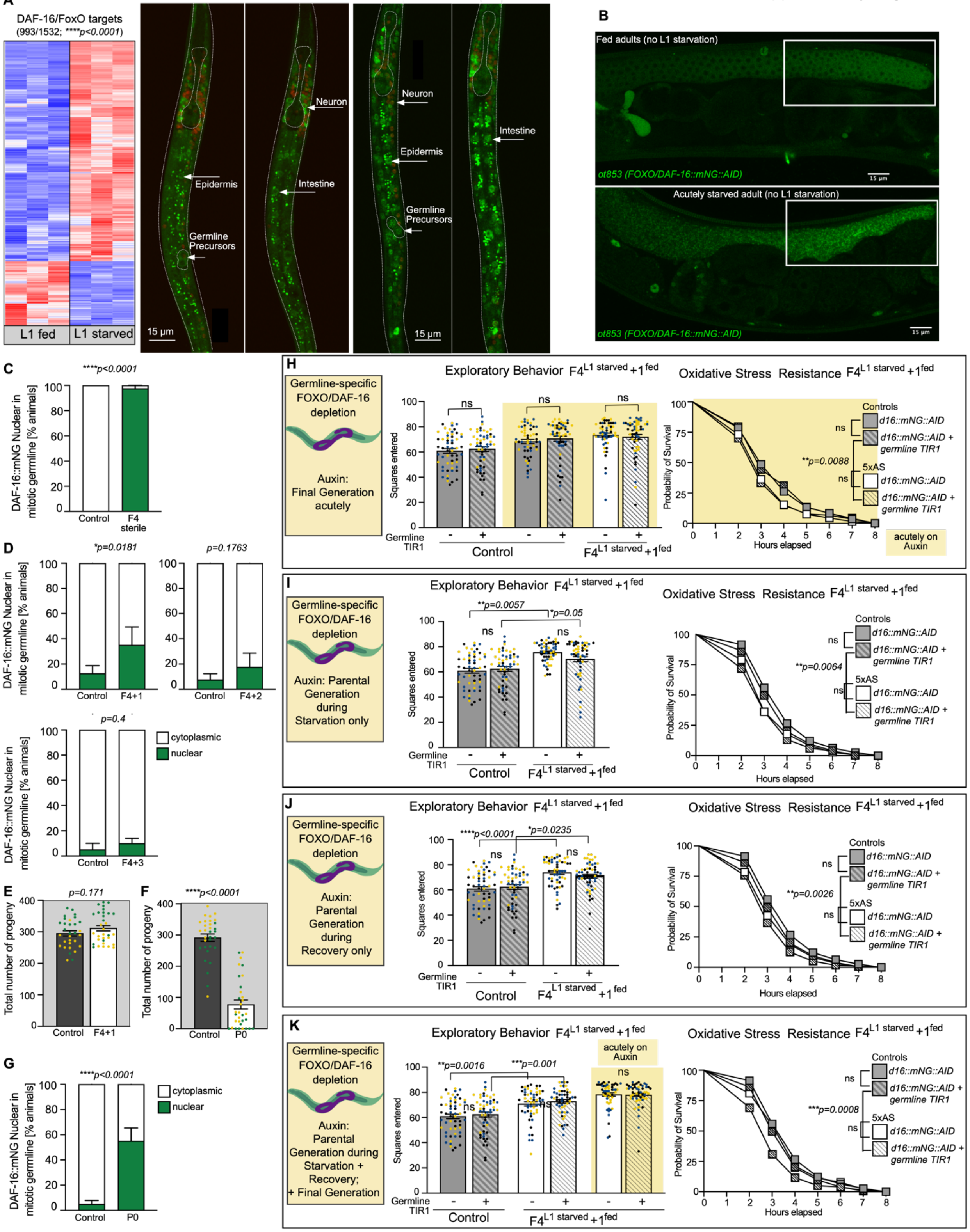

Fig. S4, DAF-16/FoxO does not act directly in the germline to mediate metabolic programming across generations, related to Fig. 5

(A) Acute starvation in control animals results in overall upregulation of class 1 IIS downstream target genes. Values of differentially expressed genes (*adj.p<0.1*) between fed and starved L1 control animals were z-score normalized and plotted using heatmap3 using RStudio. Each row represents a single gene and each column represents a single RNA-Seq replicate. Blue=relative downregulation, red=relative upregulation in starved L1 animals. Imaging of fed and acutely starved L1 animals with an endogenously-tagged mNeonGreen DAF-16/FoxO allele (*ot853*) confirms nuclear accumulation, and thus activation, of DAF-16/FoxO::mNeonGreen across the animal, including germline precursor cells, epidermis, neurons and intestines upon starvation. Scale bar =15µm. **(B)** DAF-16/FoxO::mNG also localizes (peri)nuclearly in the mitotic germline upon acute starvation in control fed adult animals. However, the severity and subcellular distribution of DAF-16/FoxO::mNG is different in acutely starved adult compared to L1 starved, currently fed adult animals. Scale bar = 15 µm. **(C)** Nearly 100% of adult fed sterile $F4^{L1starved}$ animals display nuclear accumulation of DAF-16/FoxO::mNG in mitotic germline. **(D)** Relative nuclear accumulation of DAF-16/FoxO::mNG in mitotic germline is still significantly increased in immediate progeny, but lost in subsequent descendants of $F4^{L1starved}$. Data was collected from n=10 animals/group and 3 biological replicates and is presented as mean+/-SEM. Significance was determined by Chi-square test. Color indicates sub-cellular localization of DAF-16/FoxO in mitotic germline: White= cytoplasm; Green= Nucleus. Control and ancestrally starved animals were imaged in presence of acute auxin unlike animals displayed in Fig. 4A and S4B, which could explain slight increase in nuclear accumulation in mitotic germline even in control animals. **(E)** Impaired fertility of $F4^{L1starved}$ is not mediated across generations to $F4^{L1starved}+1^{fed}$ animals. **(F)** One round of L1 starvation is sufficient to impair fertility later in life. Data is displayed as mean +/- SEM and was collected from n=16/group across two biological replicates. Significance was determined by Mann Whitney U test. **(G)** One round of L1 starvation is sufficient to induce DAF-16/FoxO nuclear accumulation in mitotic germline in adult animals. Data was collected from n=10 animals/group and 3 biological replicates and is presented as mean+/-SEM. Significance was determined by Chi-square test. **(H-K)** Results for exploratory behavior and oxidative stress resistance in $F4^{L1starved}+1^{fed}$ upon auxin-induced DAF-16/FoxO depletion specifically in the germline and only during indicated generation and time-points (same outline as displayed in Fig. 5C-5F for $F4^{L1starved}+3^{fed}$). Data for exploratory behavior was collected from n=20/group across 3 biological replicates per condition and displayed as mean +/-SEM. Statistical significance was determined using One Way ANOVA with posthoc Tukey. For oxidative stress resistance, the combined data of 3 independent biological replicates per group and condition with n=70-103/replicate is displayed. Statistical significance was determined by Log-rank (Mantel-Cox test).

Supplementary Figure 5

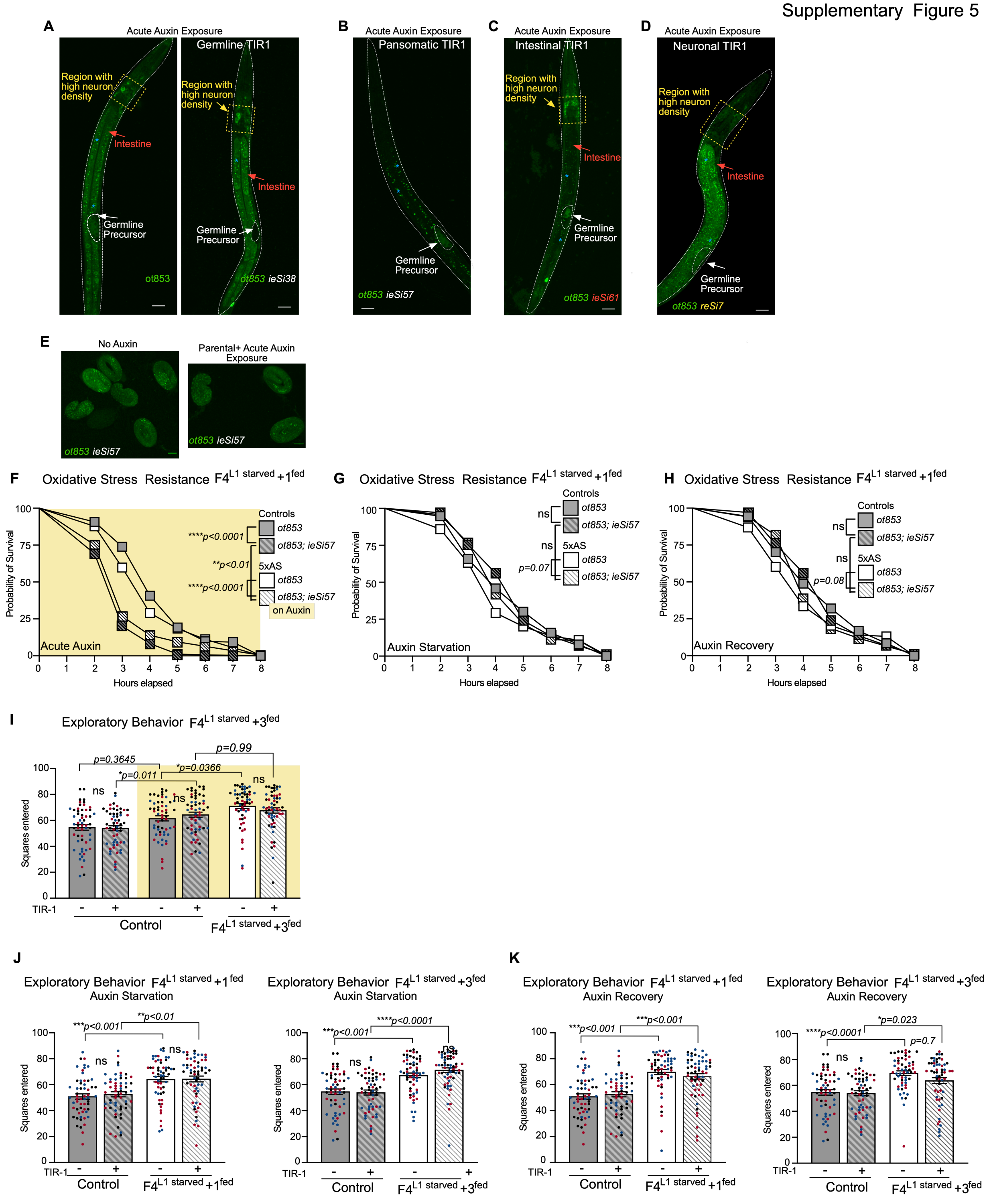

Fig. S5, Starvation-induced increase of DAF-16/FoxO activity in somatic tissues causes metabolic programming across generation, related to Fig. 6

(A-D) AID/TIR1 system efficiently and specifically depletes DAF-16/FoxO::mNG::AID from tissues of interest. DAF-16/FoxO::mNG::AID is efficiently depleted from **(A)** germline precursor cells in animals expressing TIR1 under the control of a germline-specific promoter (*ot853;ieSi38*), **(B)** pan-somatically in animals expressing TIR1 under control of a pan-somatic promoter (*ot853; ieSi57*), **(C)** the intestine in animals expressing TIR1 under control of intestine-specific promoter (*ot853, ieSi61*), and **(D)** pan-neuronally in animals expressing TIR1 under the control of a panneuronal-specific promoter (*ot853; reSi7*) in the presence of auxin only. Scale bar =15 µm. Animals are imaged at L2 stage under fed conditions. **(E)** Auxin exposure does not efficiently deplete DAF-16/FoxO::mNG::AID in embryos in our paradigm. Displayed are embryos expressing DAF-16/FoxO::mNG::AID and pan-somatic TIR1 (*ot853; ieSi57*) at various stages in the absence or presence of auxin. Scale bar =15 µm. F-H) Results for oxidative stress resistance upon pan-somatic depletion of DAF-16/FoxO during distinct time points are displayed as combined data from three independent biological replicates with n=80-114/replicate are displayed. Statistical significance was determined by Log-rank (Mantel-Cox test). **(F)** Acute pan-somatic DAF-16/FoxO depletion decreased oxidative stress in control and $F4^{L1starved}+1^{fed}$ animals. **(G)** Pan-somatic DAF-16/FoxO depletion during L1 starvation in the parental generation reverted decreased oxidative stress resistance in $F4^{L1starved}+1^{fed}$ (*ot853; ieSi57* control vs *ot853; ieSi57* $F4^{L1starved}+1^{fed}$ animals not significant). **(H)** Pan-somatic DAF-16/FoxO depletion during recovery phase in the parental generation reverted decreased oxidative stress resistance in $F4^{L1starved}+1^{fed}$ (*ot853; ieSi57* control vs *ot853; ieSi57* $F4^{L1starved}+1^{fed}$ animals not significant). **(I)** Acute pan-somatic depletion of DAF-16/FoxO was insufficient to increase exploration in either control or ancestrally starved animals. **(J)** Pan-somatic DAF-16/FoxO depletion during L1 starvation or **(K)** recovery phase in parental generation does not revert increased exploratory behavior inter- ($F4^{L1starved}+1^{fed}$) or transgenerationally ($F4^{L1starved}+3^{fed}$). Data was obtained from n=20/group and 3 independent biological replicates and is displayed as mean+/-SEM. Statistical significance was determined using One Way ANOVA with posthoc Tukey.

Supplementary Figure 6

A

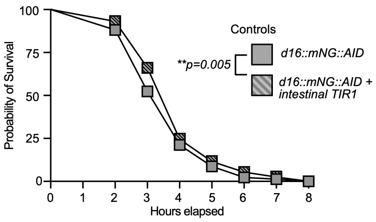

Fig. S6, Intestinal TIR1- and DAF-16/FoxO::mNG::AID expressing strain displays slight increase in oxidative stress resistance even in absence of auxin, related to Fig. 7

(A) Oxidative stress resistance is significantly increased in animals expressing DAF-16/FoxO::mNG::AID and intestine-specific TIR1 (*ot853;ieSi61*) compared to animals expressing DAF-16/FoxO::mNG::AID (*ot853)* animals even in the absence of auxin. Data is displayed as combined data from three independent biological replicates with n=80-100/replicate. Statistical significance was determined by Log-rank (Mantel-Cox test).

A

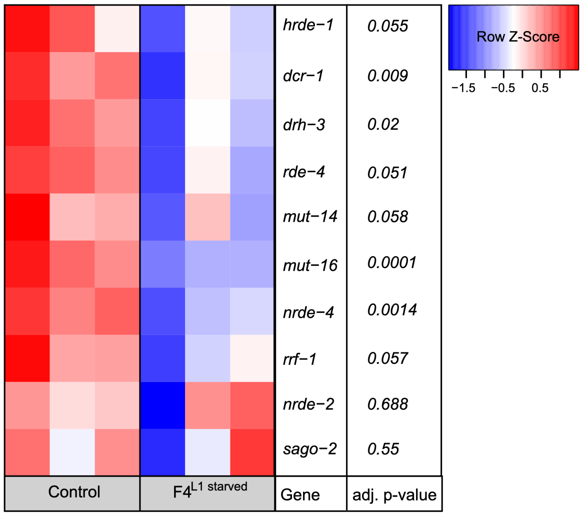
hrde−1
0.055
dcr−1
0.009
drh−3
0.02
rde−4
0.051
mut−14
0.058
mut−16
0.0001
nrde−4
0.0014
rrf−1
0.057
nrde−2
0.688
sago−2
0.55
Control
F4 L1 starved
Gene
adj. p-value
Row Z-Score
−1.5
−0.5
0.5

Fig. S7, A large subset of genes encoding for critical factors involved in the biogenesis and function of small RNAs are “class 2” downstream targets of IIS, related to Fig. 8

(A) Transcriptome analysis between control and fertile $F4^{L1starved}$ revealed that genes encoding for critical factors involved in biogenesis and function of small RNAs displayed relative downregulation in fertile $F4^{L1starved}$ compared to control animals at day 1 of adulthood under fed conditions. Values of genes between fed and starved L1 control animals were z-score normalized and plotted using heatmap3 using RStudio. Each row represents a single gene and each column represents a single RNA-Seq replicate. Blue=relative downregulation, red=relative upregulation. Adjusted p-values were determined by Wald test with subsequent Benjamini and Hochberg correction using the DESeq2 package *(97)*.

Data S1. (Separate file)

Data S1: Transcriptome Analysis Control vs F4+3 – All Genes and Conditions Related to Fig. 2

Data S2: Significantly Expressed Genes Control vs F4+3 under fed conditions Related to Fig. 2A, 2B

Data S3: Significantly Expressed Genes Control vs F4+3 under starved conditions Related to Fig. 2A, 2B

Data S4: Enrichment Differentially Expressed Genes Control vs F4+3 Fed for class1 IIS genes Related to Fig. 2E

Data S5: Enrichment Differentially Expressed Genes Control vs F4+3 Fed for class2 IIS genes Related to Fig. 2E

Data S6: Transcriptome Analysis Parental Control vs F4 – All Genes and Groups Related to Fig. 4

Data S7: Differentially expressed Genes Parental Control vs F4 fertile Related to Fig. 4C

Data S8: Enrichment Analysis Parental Control vs F4 fertile– DAF16/FoxO class1 Related to Fig. 4D

Data S9: Enrichment Analysis Parental Control vs F4 fertile– TGFb Related to Fig. 4D

Data S10: Enrichment Analysis Parental Control vs F4 fertile– 5HT_tph1mut Related to Fig. 4D

Data S11: Enrichment Analysis Parental Control vs F4 fertile– AMPK_aak2mutdown Related to Fig. 4D

Data S12: Enrichment Analysis F4 fertile vs F4 sterile – DAF16/FoxO class1 Related to Fig. S3
